# Human adipose-derived stromal/stem cells expressing doublecortin improve cartilage repair in rabbits and monkeys

**DOI:** 10.1038/s41536-021-00192-6

**Published:** 2021-11-30

**Authors:** Dongxia Ge, Michael J. O’Brien, Felix H. Savoie, Jeffrey M. Gimble, Xiying Wu, Margaret H. Gilbert, Gabrielle L. Clark-Patterson, Jason D. Schuster, Kristin S. Miller, Alun Wang, Leann Myers, Zongbing You

**Affiliations:** 1 Department of Structural and Cellular Biology, Tulane University School of Medicine, New Orleans, LA USA; 2 Department of Orthopaedic Surgery, Tulane University School of Medicine, New Orleans, LA USA; 3 LaCell LLC and Obatala Sciences Inc., New Orleans, LA USA; 4 Tulane Center for Stem Cell Research and Regenerative Medicine, Tulane University School of Medicine, New Orleans, LA USA; 5 John W. Deming Department of Medicine, Tulane University School of Medicine, New Orleans, LA USA; 6 Department of Surgery, Tulane University School of Medicine, New Orleans, LA USA; 7 Tulane National Primate Research Center, Tulane University, New Orleans, LA USA; 8 Department of Biomedical Engineering, Tulane University, New Orleans, LA USA; 9 Department of Pathology and Laboratory Medicine, Tulane University School of Medicine, New Orleans, LA USA; 10 Department of Biostatistics and Data Science, Tulane University School of Public Health and Tropic Medicine, New Orleans, LA USA; 11 Tulane Cancer Center and Louisiana Cancer Research Consortium, Tulane University School of Medicine, New Orleans, LA USA; 12 Tulane Center for Aging, Tulane University School of Medicine, New Orleans, LA USA; 13 Southeast Louisiana Veterans Health Care System, New Orleans, LA USA

**Keywords:** Regenerative medicine, Mesenchymal stem cells

## Abstract

Localized cartilage lesions in early osteoarthritis and acute joint injuries are usually treated surgically to restore function and relieve pain. However, a persistent clinical challenge remains in how to repair the cartilage lesions. We expressed doublecortin (DCX) in human adipose-derived stromal/stem cells (hASCs) and engineered hASCs into cartilage tissues using an in vitro 96-well pellet culture system. The cartilage tissue constructs with and without DCX expression were implanted in the knee cartilage defects of rabbits (*n* = 42) and monkeys (*n* = 12). Cohorts of animals were euthanized at 6, 12, and 24 months after surgery to evaluate the cartilage repair outcomes. We found that DCX expression in hASCs increased expression of growth differentiation factor 5 (GDF5) and matrilin 2 in the engineered cartilage tissues. The cartilage tissues with DCX expression significantly enhanced cartilage repair as assessed macroscopically and histologically at 6, 12, and 24 months after implantation in the rabbits and 24 months after implantation in the monkeys, compared to the cartilage tissues without DCX expression. These findings suggest that hASCs expressing DCX may be engineered into cartilage tissues that can be used to treat localized cartilage lesions.

## Introduction

In the United States, arthritis is the most common cause of disability^1^. The most common form of arthritis is osteoarthritis and other forms include rheumatoid arthritis, gout, lupus, and fibromyalgia. Osteoarthritis (OA) affects over 32.5 million US adults according to the Centers for Disease Control and Prevention. OA has a complex and multifactorial (genetic, biological, and biomechanical) pathogenesis. About 20% to more than 50% of patients who suffer acute cartilage injuries may develop post-traumatic OA^2^. Articular cartilage lesions are common after acute joint trauma, which are usually treated clinically with microfracture, mosaicplasty (osteochondral autograft transfer), and autologous chondrocyte transplantation that is still being modified for improvement^3,4^. New strategies based on mesenchymal stem cells are being actively tested in humans and animals^5,6^.

Doublecortin (DCX) is a microtubule-binding protein that stabilizes microtubules and causes bundling^7^, which was originally found in neuronal precursor cells and immature neurons during neurogenesis^8–10^. Later, DCX was found in osteochondral precursors and immature articular chondrocytes^11,12^. DCX has been widely used as a marker of articular chondrocytes^11,13–19^ and articular chondrocyte lineage in chondrogenesis^20,21^; however, its functional role in chondrogenesis is not clear. DCX expression overlaps that of Sex Determining Region Y-Box Transcription Factor 9 (Sox9), which is expressed by osteochondral precursor cells in the developing limb mesenchyme^12,22^. Studies on *Dcx*-reporter mice found that *Dcx* is initially expressed throughout the limb mesenchyme and is maintained within the joint interzone but lost in the adjacent regions of the cartilaginous anlagen^12^. The fates of the joint interzone and adjacent cartilaginous anlagen cells (identified as endochondral chondrocytes) are distinct in that the joint interzone cells differentiate into articular chondrocytes, but the endochondral chondrocytes undergo proliferation, hypertrophy, and apoptosis. Thus, it is speculated that DCX may play an important role in directing osteochondral precursors to differentiate into articular chondrocytes. Our previous in vitro study found that ectopic expression of DCX in human adipose-derived stromal/stem cells (hASCs) increased expression of growth differentiation factor 5 (GDF5) and matrilin 2 (MATN2) in the cartilage tissues engineered from 14-day pellet cultures^23^. Since GDF5 and MATN2 expression is restricted to articular chondrocytes^20,24,25^, the finding suggests that DCX expression leads to formation of a cartilage that is more like bona fide articular cartilage than the cartilage formed without DCX expression. Thus, we hypothesized that DCX expression promotes differentiation of hASCs into articular chondrocytes and tissue engineered cartilage tissues with DCX expression may provide better cartilage repair outcomes than those without DCX expression. To test this hypothesis, we implanted the pellet culture-engineered cartilage tissues (with or without DCX expression) into knee cartilage defects in 42 rabbits and 12 rhesus macaques and followed the animals up to 24 months. We demonstrate that cartilage tissues engineered from hASCs expressing DCX yield significantly better cartilage repair outcomes than cartilage tissues engineered from hASCs without DCX expression. Our findings suggest that hASCs expressing DCX may be engineered into cartilage tissues that can be used to treat localized cartilage lesions.

## Results

### DCX expression in hASCs enhances “articular cartilage” phenotype of tissue engineered cartilage

**Fig. 1:**
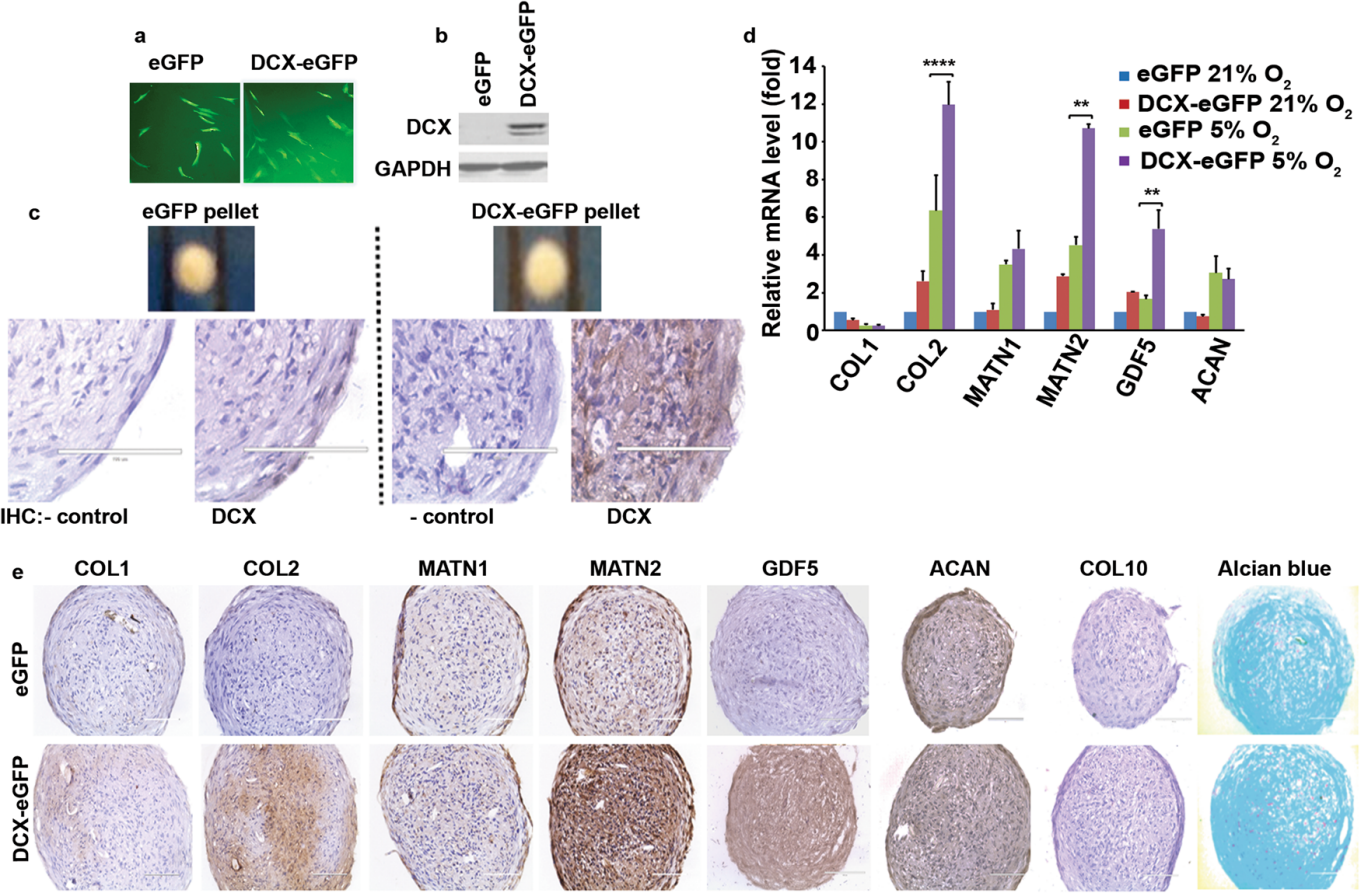
DCX expression in hASCs enhances “articular cartilage” phenotype of tissue engineered cartilage. **a** Representative photomicrographs of hASCs 48 h after transduction with HRST-eGFP and HRST-DCX-GP-eGFP lentiviruses, showing eGFP expression under a fluorescent microscope (×100 magnification). **b** Western blot analysis of DCX expression in the transduced hASCs; glyceraldehyde 3-phosphate dehydrogenase (GAPDH) served as a protein load control; DCX and GAPDH blots were derived from the same membrane; original scans are provided in Supplementary Fig. 11. **c** Representative pictures of the cartilage pellets (upper row; two dark bars are the 1-mm scale on a ruler) and IHC staining of DCX and negative control (lower row); scale bars = 100 µm. **d** qRT-PCR analysis of the mRNA levels of collagen 1 (*COL1*), collagen 2 (*COL2*), matrilin 1 (*MATN1*), matrilin 2 (*MATN2*), growth differentiation factor 5 (*GDF5*), and aggrecan (*ACAN*) in the cartilage pellets; data represent mean ± standard deviation (error bars) of three independent experiments (*n* = 3); **P < 0.01 and ****P < 0.0001. **e** Representative photomicrographs of IHC staining and Alcian blue staining; scale bars = 100 µm.

hASCs were isolated from lipoaspirate tissues gathered from elective surgical procedures and were characterized for immunophenotypes (CD29^+^CD34^+^CD73^+^CD90^+^CD105^+^CD44^low^CD45^low^) and differentiation properties (adipogenesis and osteogenesis)^26^. hASCs were transduced with replication-incompetent lentiviruses expressing enhanced green fluorescence protein (eGFP) tag (HRST-eGFP and HRST-DCX-GP-eGFP) as previously described^23^. Forty-eight hours(48 h) later, transduction was verified under a fluorescent microscope (Fig. 1a) and DCX expression was confirmed by western blot analysis (Fig. 1b)^23^. The transduced hASCs were plated into a 96-well pellet culture system for 14 days to produce large number of cartilage tissues (named as eGFP pellets or DCX-eGFP pellets). Each pellet was approximately 1 mm in diameter, and DCX expression was confirmed by immunohistochemical (IHC) staining (Fig. 1c). Compared with eGFP pellets, DCX-eGFP pellets expressed higher levels of collagen 2 (*COL2*), matrilin 2 (*MATN2*), and *GDF5* mRNAs under normoxia (21% O_2_) and hypoxia (5% O_2_) culture conditions (Fig. 1d). The mRNA levels of collagen 1 (*COL1*), matrilin 1 (*MATN1*), and aggrecan (*ACAN*) were comparable (Fig. 1d). IHC staining showed that COL2, MATN2, and GDF5 protein levels were increased in DCX-eGFP pellets, compared with eGFP pellets (Fig. 1e). Type X collagen (COL10) staining was negative in both groups (Fig. 1e). Alcian blue staining did not show any obvious difference between eGFP pellets and DCX-eGFP pellets (Fig. 1e), which is consistent with the comparable levels of ACAN in both groups. Since GDF5 and MATN2 expression is restricted to articular chondrocytes^20,24,25^, increased expression of GDF5 and MATN2 indicates that DCX-eGFP pellets are more like “articular cartilage” than eGFP pellets. Since our data (Fig. 1d, e) and other studies^27–29^ indicate that hypoxia enhances chondrogenic differentiation of mesenchymal stem cells, we adopted the 96-well pellet culture under hypoxic (5% O_2_) conditions to produce cartilage tissues in large quantities for our animal studies.

### Tissue engineered cartilage tissues with DCX expression improve cartilage repair outcomes in rabbits

**Fig. 2:**
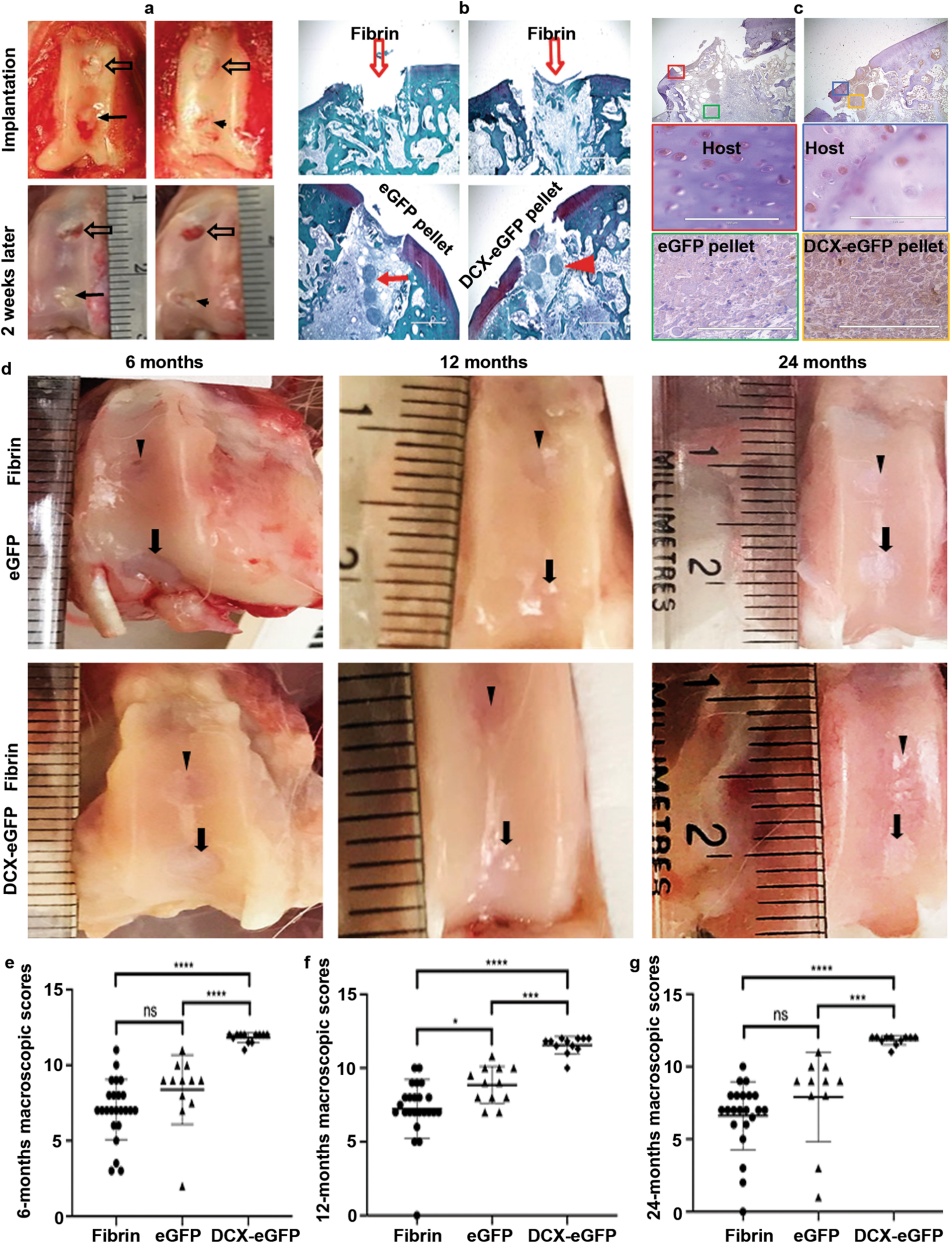
Rabbit knee cartilage defect repair surgery and macroscopic evaluation of repair outcomes. **a** Representative pictures of cartilage defects in the rabbit knee femoral grooves repaired with fibrin (open arrows), eGFP pellets (solid arrows), and DCX-eGFP pellets (arrowheads). **b** Representative photomicrographs of Safranin O-stained cartilage tissue sections at 2 weeks after implantation; open arrows, defects filled with fibrin; solid arrow, defect filled with eGFP pellets; arrowhead, defect filled with DCX-eGFP pellets; scale bars = 1000 µm. **c** Representative photomicrographs of IHC staining for DCX in the cartilage tissues at 2 weeks after implantation; upper row, ×40 magnification (scale bar = 1000 µm), the color-coded regions of interest are magnified at ×400 in the middle and lower rows (scale bars = 100 µm). **d** Representative pictures of the macroscopic views of the cartilage defects repaired; arrowheads, fibrin-filled defects; arrows, pellets-filled defects. **e** Quantitative macroscopic scores of the rabbit cartilage repair outcomes at 6 months; *n* = 24 samples for the fibrin group (combined both knees), *n* = 12 for the eGFP pellets group, and *n* = 12 for the DCX-eGFP pellets group. **f** Quantitative macroscopic scores of the rabbit cartilage repair outcomes at 12 months; *n* = 24 samples for the fibrin group (combined both knees), *n* = 12 for the eGFP pellets group, and *n* = 12 for the DCX-eGFP pellets group. **g** Quantitative macroscopic scores of the rabbit cartilage repair outcomes at 24 months; *n* = 22 samples for the fibrin group (combined both knees), *n* = 11 for the eGFP pellets group, and *n* = 11 for the DCX-eGFP pellets group. *P < 0.05, ***P < 0.001, and ****P < 0.0001; n.s. not significant.

Two cartilage defects (3.5-mm in diameter/5-mm in depth and 5-mm apart) were made in the femoral trochlear grooves of both knees in 42 New England White rabbits (retired female and male breeders). The proximal defect of each knee was filled with fibrin sealant as untreated control. The distal defect of each knee was randomized to receive implantation of either eGFP pellets (control group) or DCX-eGFP pellets (treatment group) (Fig. 2a, upper row). Two weeks after surgical implantation, five rabbits were euthanized to confirm that the cartilage pellets stayed in the defects (Fig. 2a, lower row). Safranin O-stained cartilage sections showed that the cartilage pellets stayed in the defects, while the fibrin-filled defects were empty or filled with some loose fibrous tissues (Fig. 2b). IHC staining showed that DCX-eGFP pellets in the defect and adjacent host cartilage were stained positive for DCX expression, while the eGFP pellets were stained negative (Fig. 2c). Then, cohorts of rabbits (*n* = 11–12) were euthanized at 6, 12, and 24 months after surgery. Macroscopic examination showed that the cartilage defects were mostly repaired with neocartilage in both eGFP pellets and DCX-eGFP pellets groups, and even the cartilage defects filled with fibrin were mostly covered with new tissues (Fig. 2d). To quantify the cartilage repair outcomes macroscopically, we followed the International Cartilage Repair Society (ICRS) assessment scales (Supplementary Table 1)^30–32^. We found that the macroscopic scores of DCX-eGFP pellets group were significantly higher than either eGFP pellets group or fibrin group at all time points (6, 12, and 24 months) (Fig. 2e–g and Supplementary Fig. 1). Macroscopic scores of the eGFP pellets group were higher than the fibrin group, but were only statistically significant at 12 months after surgery (Fig. 2e–g). In combination of the three time points, DCX-eGFP pellets group achieved 19/35 (54%) of the defects with Grade I (normal) and 16/35 (46%) of the defects with Grade II (nearly normal) cartilage repair, which was significantly better than either eGFP pellets group or fibrin group (Supplementary Table 2).

**Fig. 3:**
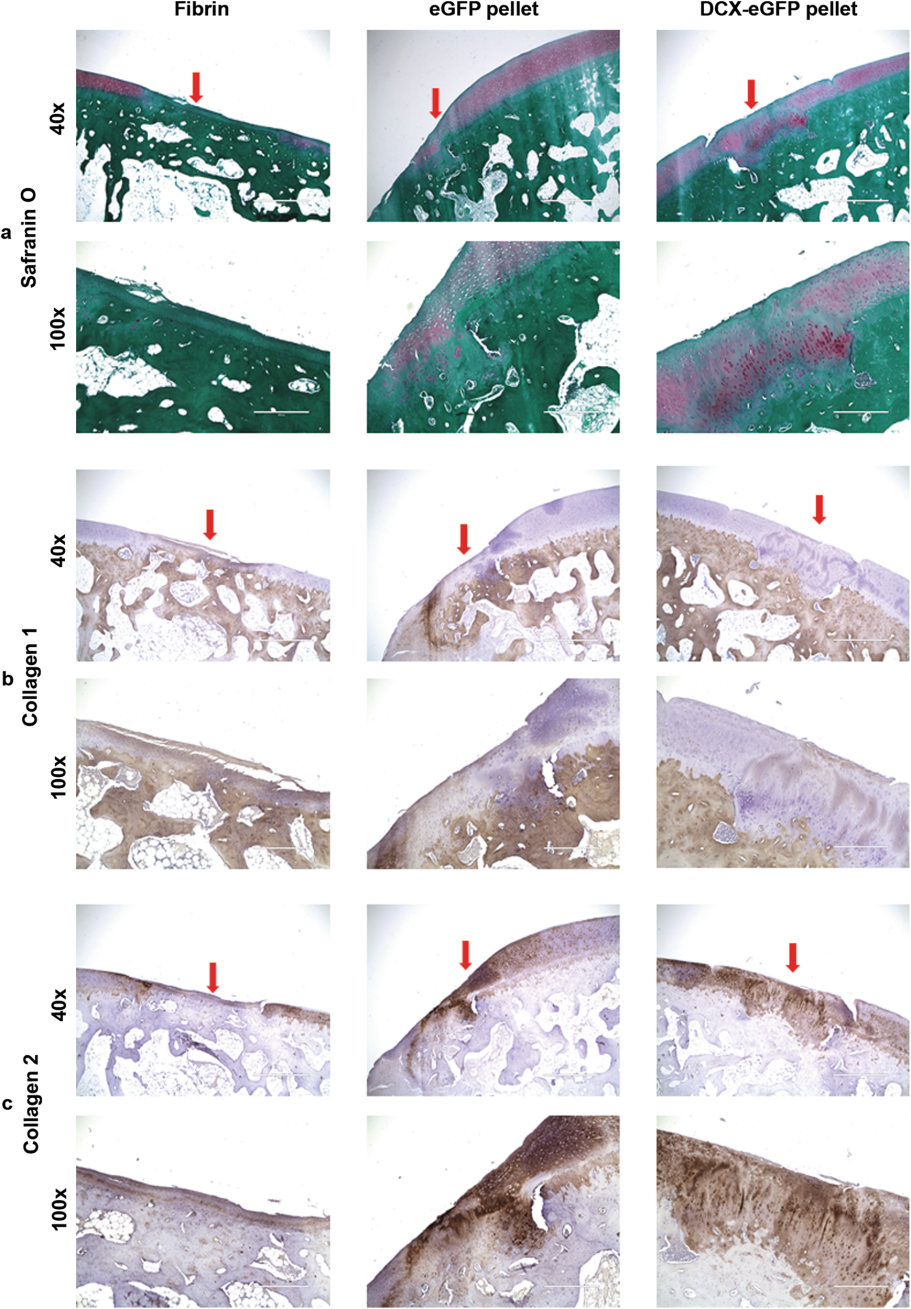
Representative photomicrographs of rabbit cartilage tissue sections at 6 months after surgery. **a** Safranin O staining. **b** Immunohistochemical staining of collagen 1. **c** Immunohistochemical staining of collagen 2. Arrows, the repaired cartilage defects. ×40 magnification, scale bars = 1000 µm. ×100 magnification, scale bars = 400 µm.

**Fig. 4:**
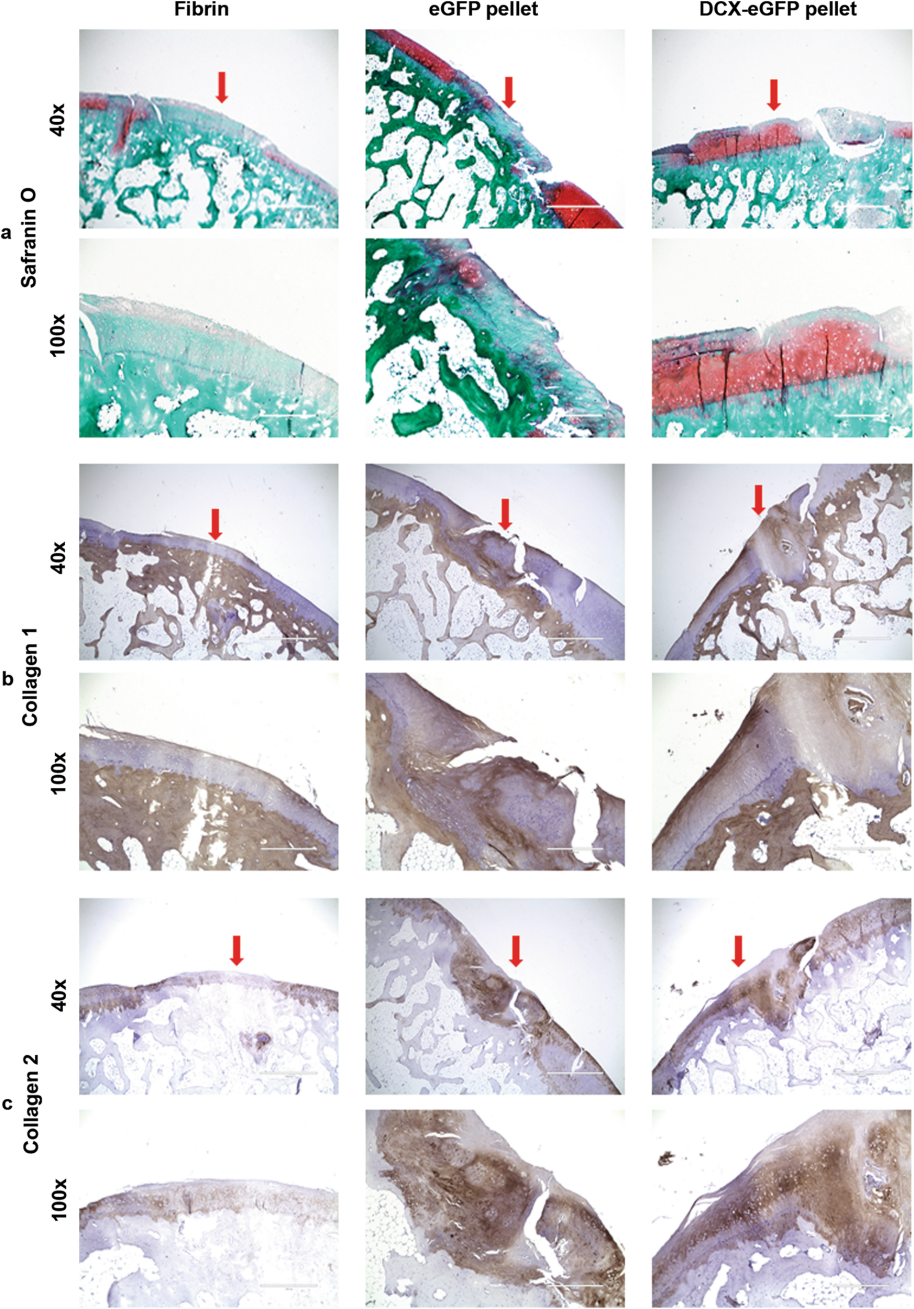
Representative photomicrographs of rabbit cartilage tissue sections at 12 months after surgery. **a** Safranin O staining. **b** Immunohistochemical staining of collagen 1. **c** Immunohistochemical staining of collagen 2. Arrows, the repaired cartilage defects. ×40 magnification, scale bars = 1000 µm. ×100 magnification, scale bars = 400 µm.

**Fig. 5:**
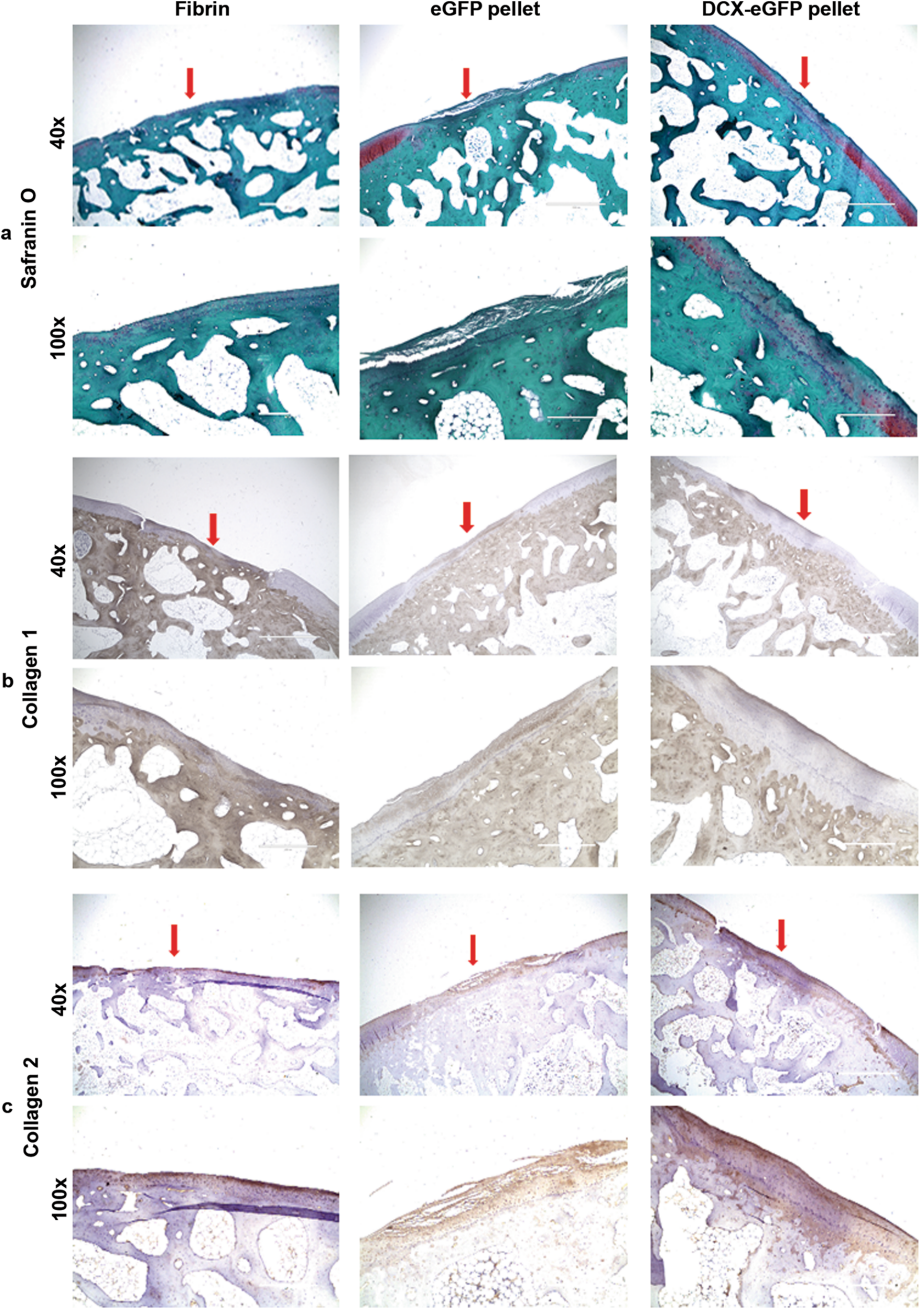
Representative photomicrographs of rabbit cartilage tissue sections at 24 months after surgery. **a** Safranin O staining. **b** Immunohistochemical staining of collagen 1. **c** Immunohistochemical staining of collagen 2. Arrows, the repaired cartilage defects. ×40 magnification, scale bars = 1000 µm. ×100 magnification, scale bars = 400 µm.

**Fig. 6:**
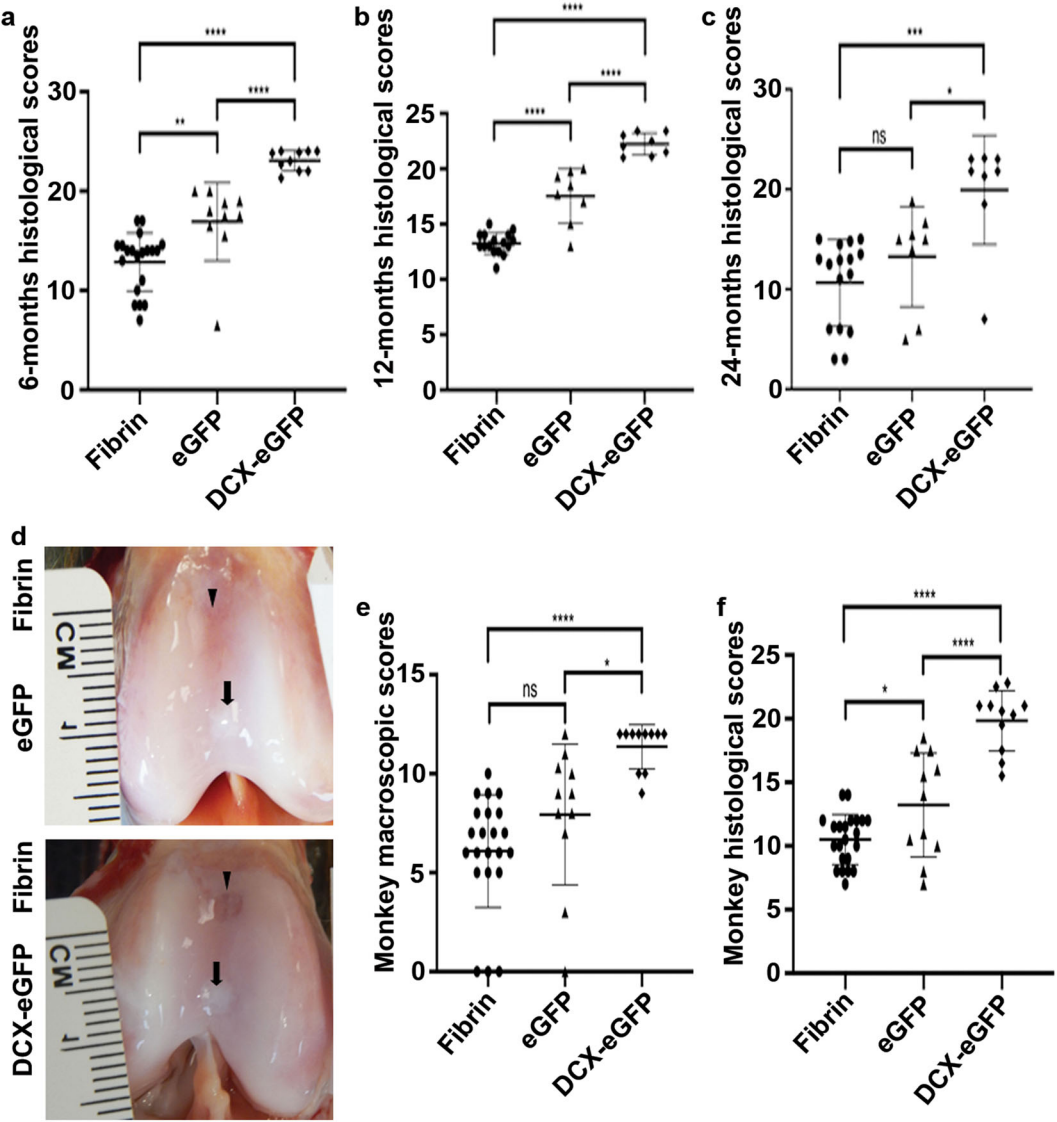
Histological evaluation and/or macroscopic evaluation of rabbit and monkey cartilage repair outcomes. **a** Histological scores of rabbit cartilage repair at 6 months after surgery; *n* = 20 samples for the fibrin group (combined both knees), *n* = 10 for the eGFP pellets group and *n* = 10 for the DCX-eGFP pellets group. **b** Histological scores of rabbit cartilage repair at 12 months after surgery; *n* = 16 samples for the fibrin group (combined both knees), *n* = 8 for the eGFP pellets group, and *n* = 8 for the DCX-eGFP pellets group. **c** Histological scores of rabbit cartilage repair at 24 months after surgery; *n* = 16 samples for the fibrin group (combined both knees), *n* = 8 for the eGFP pellets group, and *n* = 8 for the DCX-eGFP pellets group. **d** Representative pictures of the macroscopic views of the monkey cartilage defects repaired; arrowheads, fibrin-filled defects; arrows, pellets-filled defects. **e** Macroscopic scores of monkey cartilage repair at 24 months after surgery; *n* = 22 samples for the fibrin group (combined both knees), *n* = 11 for the eGFP pellets group, and *n* = 11 for the DCX-eGFP pellets group. **f** Histological scores of monkey cartilage repair at 24 months after surgery; *n* = 22 samples for the fibrin group (combined both knees), *n* = 11 for the eGFP pellets group, and *n* = 11 for the DCX-eGFP pellets group. *P < 0.05, **P < 0.01, ***P < 0.001, and ****P < 0.0001; n.s. not significant.

To examine the histological structure of the cartilage repair, we stained the cartilage tissue sections for Safranin O, collagen 1, and collagen 2. As shown in Fig. 3, at 6 months after surgery, the fibrin-filled cartilage defects were mostly repaired with fibrous tissues and stained dark green with Safranin O, consistent with a non-robust extracellular matrix expression (Fig. 3a). Both eGFP pellets- and DCX-eGFP pellets-filled cartilage defects were mostly repaired with neocartilage and stained mostly red with Safrain O, consistent with robust collagen 2 and proteoglycan expression (Fig. 3a). IHC staining of collagen 1 showed that fibrin-filled defects were stained strongly positive, whereas DCX-eGFP pellets-filled defects were stained mostly negative with faint positive regions and eGFP pellets-filled defects were stained with a mixture of negative and positive regions (Fig. 3b). IHC staining of collagen 2 showed that fibrin-filled defects were stained almost negative, whereas DCX-eGFP pellets-filled defects were stained strongly positive while eGFP pellets-filled defects were stained patchily positive (Fig. 3c). These staining patterns were similarly found in the cartilage tissue sections at 12 months (Fig. 4) and 24 months (Fig. 5) after surgery. To quantify the histological features, we adopted the modified ICRS visual histological assessment criteria (Supplementary Table 3)^33,34^. We found that the histological scores of the DCX-eGFP pellets group were significantly higher than either eGFP pellets group or fibrin group at all time points (6, 12, and 24 months) (Fig. 6a–c and Supplementary Fig. 2). Histological scores of the eGFP pellets group were higher than the fibrin group, which were statistically significant at 6 and 12 months after surgery but not statistically significant at 24 months after surgery (Fig. 6a–c). To evaluate the mechanical properties of the repaired cartilages, we performed a pilot unconfined compression test on the cartilage samples from 3 rabbits at 12 months and 3 rabbits at 24 months after surgery. We found that the stiffness (represented as equilibrium modulus) of the repaired cartilage was not statistically different among the groups tested (Supplementary Fig. 3).

### Tissue engineered cartilage tissues with DCX expression improve cartilage repair outcomes in monkeys

As described in the rabbit models, cartilage defects were made and repaired in the femoral trochlear grooves of both knees in 12 rhesus macaques (6 males and 6 females, average age 7.24 ± 1.37 years). Twenty-four months after surgery, all monkeys were euthanized except one who was euthanized at 12 months after surgery due to illness. Macroscopic examination showed that both the proximal and distal cartilage defects were repaired after 24 months (Fig. 6d). We quantified the cartilage repair outcomes macroscopically according to the ICRS assessment scales (Supplementary Table 1). We found that the macroscopic score of the DCX-eGFP pellets group was significantly higher than either eGFP pellets group or fibrin group (Fig. 6e). Macroscopic score of the eGFP pellets group was higher than the fibrin group, but was not statistically significant (Fig. 6e).

**Fig. 7:**
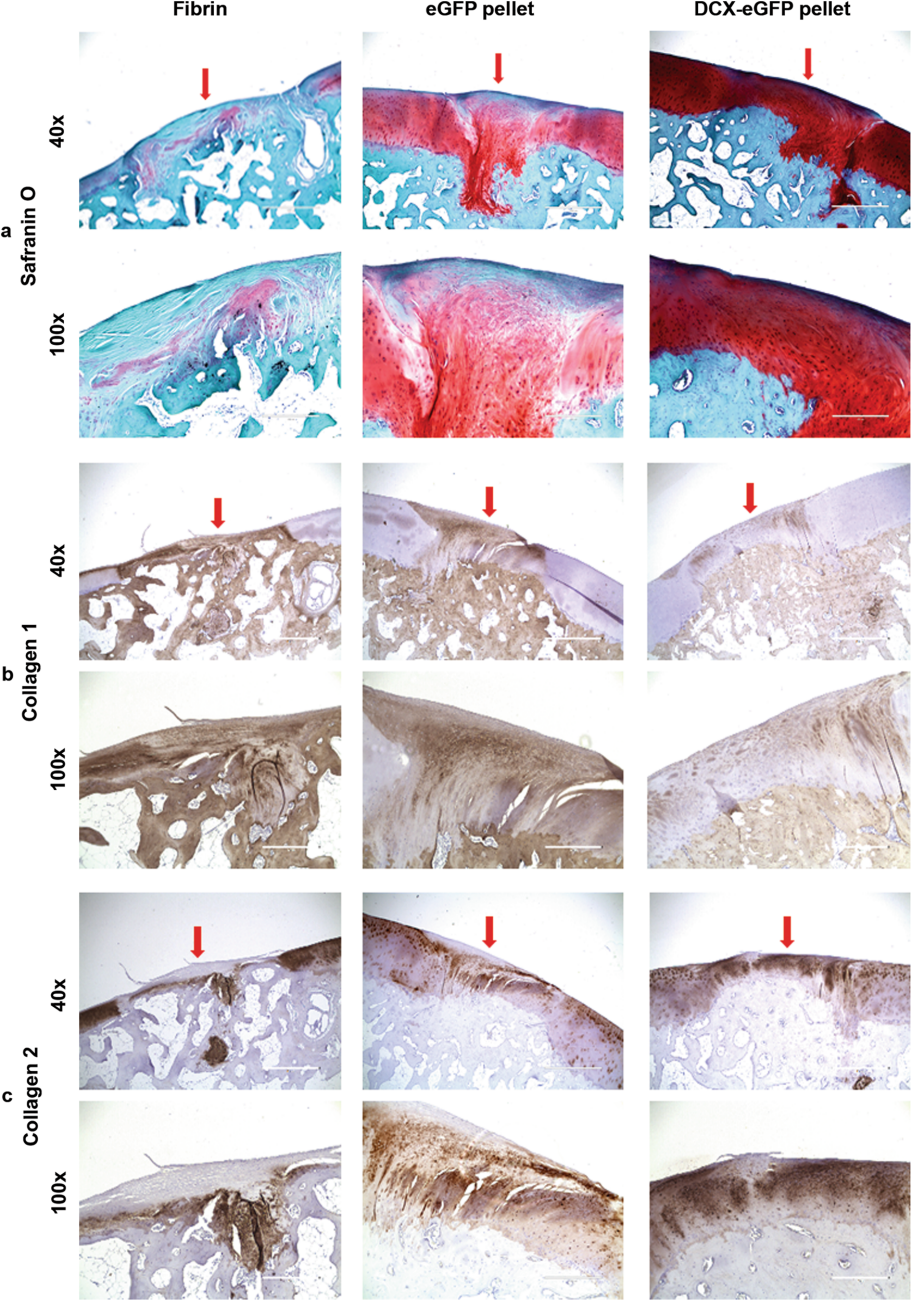
Representative photomicrographs of monkey cartilage tissue sections at 24 months after surgery. **a** Safranin O staining. **b** Immunohistochemical staining of collagen 1. **c** Immunohistochemical staining of collagen 2. Arrows, the repaired cartilage defects. ×40 magnification, scale bars = 1000 µm. ×100 magnification, scale bars = 400 µm.

To examine the histological structure of the cartilage repair, we stained the cartilage tissue sections for Safranin O, collagen 1, and collagen 2. As shown in Fig. 7, the fibrin-filled cartilage defects were mostly repaired with fibrous tissues and stained green with Safranin O, but small areas adjacent to the subchondral bone showed red staining (Fig. 7a). The eGFP pellets-filled cartilage defects were mostly repaired with neocartilage and stained red with Safrain O, but with some green staining indicating fibrous tissues in nature (Fig. 7a). The DCX-eGFP pellets-filled cartilage defects were mostly stained red (Fig. 7a). IHC staining of collagen 1 showed that fibrin-filled defects were stained strongly positive, whereas DCX-eGFP pellets-filled defects were stained mostly negative with faint positive regions and eGFP pellets-filled defects were stained mostly positive (Fig. 7b). IHC staining of collagen 2 showed that fibrin-filled defects were stained almost negative, but with positive staining adjacent to the bone where Safranin O staining was red (Fig. 7c). In contrast, DCX-eGFP pellets-filled defects were stained strongly positive while eGFP pellets-filled defects were stained patchily positive (Fig. 7c). We quantified the histological features according to the modified ICRS visual histological assessment criteria (Supplementary Table 3). We found that the histological score of DCX-eGFP pellets group was significantly higher than either eGFP pellets group or fibrin group (Fig. 6f). Histological score of the eGFP pellets group was significantly higher than the fibrin group (Fig. 6f). Of note, significant differences among the groups were found in all categories of ICRS visual histological assessment criteria except the cell population viability, subchondral bone, and cartilage mineralization in both rabbits and monkeys (Supplementary Table 4). There were no significant differences between male and female rabbits and monkeys in either macroscopic scores or histological scores (Supplementary Table 5).

### DCX expression enhances GDF5 expression to promote articular chondrocyte differentiation of hASCs and host bone marrow-derived mesenchymal stem cells

**Fig. 8:**
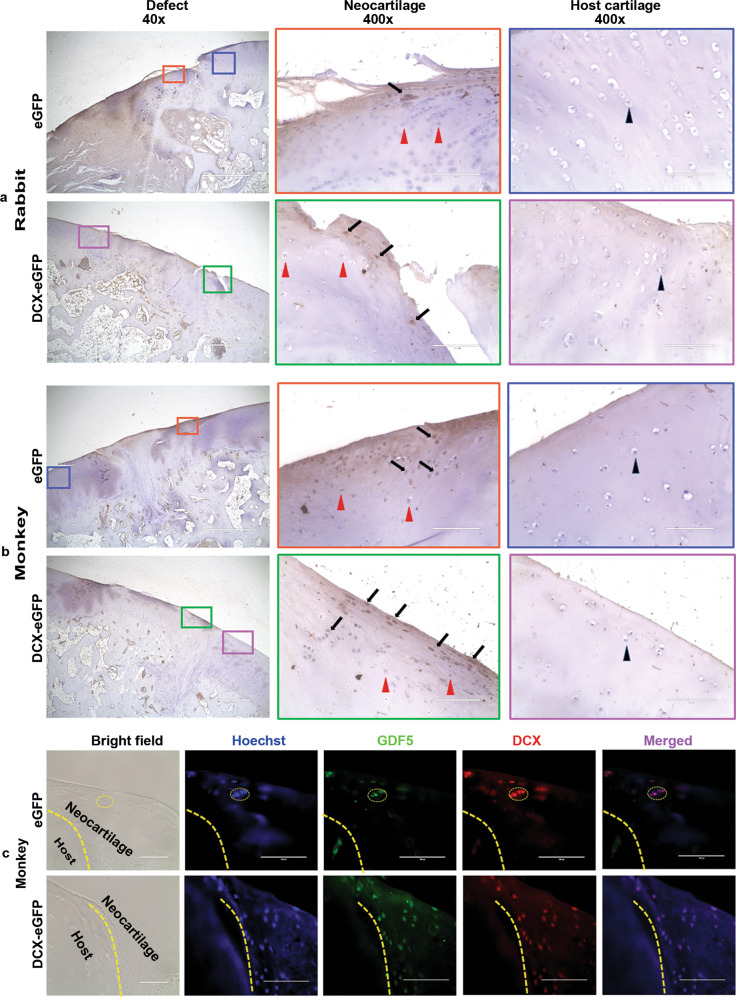
Immunohistochemical staining and immunofluorescent double staining of the cartilage tissues. **a** Representative photomicrographs of IHC staining of eGFP in the rabbit cartilage tissues at 6 months after surgery; color-coded regions of interest in the ×40 field (scale bars = 1000 µm) were magnified under ×400 (scale bars = 100 µm); arrows, eGFP-positive chondrocytes; red arrowheads, eGFP-negative chondrocytes in the neocartilage; black arrowheads, eGFP-negative chondrocytes in the host cartilage. **b** Representative photomicrographs of IHC staining of eGFP in the monkey cartilage tissues at 24 months after surgery; color-coded regions of interest in the ×40 field (scale bars = 1000 µm) were magnified under ×400 (scale bars = 100 µm); arrows, eGFP-positive chondrocytes; red arrowheads, eGFP-negative chondrocytes in the neocartilage; black arrowheads, eGFP-negative chondrocytes in the host cartilage. **c** Representative photomicrographs of immunofluorescent double staining of GDF5 and DCX in the monkey cartilage tissues; dotted lines outline the boundaries between the neocartilage and host cartilage; many GDF5/DCX double-positive chondrocytes are shown in the neocartilage of the DCX-eGFP pellets group and a cluster of GDF5/DCX double-positive chondrocytes are shown in the neocartialge of the eGFP pellets group (dotted cycle); scale bars = 100 µm.

To verify if hASCs-differentiated chondrocytes engrafted in the neocartilage grown in the cartilage defects, we performed IHC staining against eGFP that was only expressed by the transduced hASCs. We found that eGFP-positive chondrocytes were scattered in the neocartilage of the rabbits in both eGFP pellets group and DCX-eGFP pellets group while eGFP-negative chondrocytes were also present in the neocartilage at 6 months after surgery (Fig. 8a). In contrast, only eGFP-negative chondrocytes were present in the adjacent host cartilage (Fig. 8a). Similar findings were observed in the rabbits at 12 and 24 months after surgery (Supplementary Fig. 4) and in the monkeys at 24 months after surgery (Fig. 8b). The eGFP-negative chondrocytes in the neocartilage might originate from two sources: (1) hASCs not transduced by the lentiviruses because we did not purify the transduced hASCs due to high cell death rates during the lengthy flow cytometry cell sorting procedure and/or (2) host bone marrow-derived mesenchymal stem cells. To distinguish between the two possible sources, we performed additional staining using biomarkers (Ku80, human nuclei, and human mitochondria) that identify the cells of human origin^35,36^. We found that there were both Ku80-positive and Ku80-negative chondrocytes in the neocartilage of the monkeys in both eGFP pellets group and DCX-eGFP pellets group, while all chondrocytes in the adjacent host cartilage were Ku80-negative (Supplementary Fig. 5a). Similarly, anti-human nuclei and mitochondria antibody staining showed both positive and negative chondrocytes in the neocartilage, but only negative chondrocytes in the adjacent host cartilage (Supplementary Fig. 5b, c). Likewise, we found similar staining results in the rabbit cartilage tissues (Supplementary Fig. 6). These findings suggest that the chondrocytes residing in the neocartilage might originate from both the implanted hASCs-derived chondrocytes and the host endogenous bone marrow stem cells-derived chondrocytes.

Since we have demonstrated that DCX-expressing joint interzone cells differentiate into articular chondrocytes (but not synovium, ligaments, or menisci)^11,12^ and other investigators have shown that GDF5-expressing progenitor cells give rise to articular chondrocytes, ligaments, and synovium^37^, it is reasonable to speculate that articular chondrocytes should simultaneously express both DCX and GDF5. Indeed, immunofluorescent double staining showed co-expression of DCX and GDF5 in the articular chondrocytes in the monkey neocartilage (Fig. 8c) and rabbit neocartilage (Supplementary Fig. 7a). Of note, the number of DCX/GDF5 double-positive chondrocytes in the DCX-eGFP pellets group was significantly more than that of the eGFP pellets group (Supplementary Fig. 7b, c). Further, addition of recombinant GDF5 in the chondrogenic medium of the pellet culture system significantly increased mRNA expression of *DCX* and *COL2* in the cartilage pellets (Supplementary Fig. 7d). Wnt9A (also known as Wnt-14) has been shown to play a pivotal role in inducing synovial joint formation^38^; however, recombinant Wnt9A slightly inhibited *DCX* expression and antagonized GDF5’s induction of *DCX* expression (Supplementary Fig. 7d).

We did not observe any hypertrophy of the neocartilage tissues as shown by negative type X collagen staining (Supplementary Fig. 8). Since we implanted human ASCs and ASC-derived cartilage into rabbits and monkeys, we studied host immune responses to the xenogenic transplantation using CD45 staining. CD45 (protein tyrosine phosphatase, receptor type C, also known as leukocyte common antigen) is a type I transmembrane protein that is present on all differentiated hematopoietic cells (except erythrocytes and plasma cells). We did not observe obvious CD45^+^ immune cell infiltration at the boundary of neocartilage and bone or inside the neocartilage (Supplementary Fig. 9). Further, we did not find any difference in the numbers of host-derived cells in the rabbit and monkey neocartilages between DCX-eGFP pellets group and eGFP pellets group (Supplementary Fig. 10).

## Discussion

During embryonic joint development, mesenchymal condensation creates an endochondral anlage containing endochondral chondrocytes^39–41^. At the presumptive joint site, endochondral chondrocytes de-differentiate into joint interzone cells^42^. The joint interzone contains three layers: a central intermediate layer surrounded by two chondrogenous layers. The joint interzone develops into a joint through cavitation, in which the two chondrogenous layers re-differentiate into articular chondrocytes^39–41^. Although the cellular morphology and major extracellular matrix (COL2 and ACAN) produced by endochondral and articular chondrocytes appear similar, endochondral chondrocytes undergo proliferation, hypertrophy, and apoptosis, whereas articular chondrocytes display permanent residence in situ. The distinct fates of endochondral and articular chondrocytes seem to be controlled by distinct gene expression profiles^43^. However, the current paradigm for an in vitro tissue engineered cartilage model relies on high-density culture (pellet or micro-mass)^44,45^, which mimics mesenchymal condensation. The resulting cartilage belongs to hyaline cartilage, but its endochondral nature has raised serious concerns because it undergoes premature hypertrophy^46^. None of the cartilage generated by the current tissue-engineering protocol has been proven useful in the treatment of localized cartilage lesions. Thus, a critical challenge remains to discover how to engineer bona fide articular cartilage for clinical translation. In the present study, we found that DCX expression in hASCs led to formation of cartilage tissues with high levels of GDF5 and MATN2 expression, two markers of articular chondrocytes. This finding is in line with our previous study^23^. We found that the cartilage tissues with DCX expression produced significantly better repair outcomes than the ones without DCX expression in both rabbit and monkey knee cartilage defect models. Of note, we did not observe any obvious hypertrophy of the neocartilage or CD45^+^ immune cell infiltration at the osteochondral boundary or within the neocartilage. This lack of immune rejection may be due to that the dense matrix produced by chondrocytes inhibits lymphocyte migration, preventing immune detection rendering them “antigen sequestered”, and mesenchymal stem cells may have unique immunomodulatory properties including their ability to reduce immune cell infiltration and to modulate inflammation^47^. Articular cartilage is considered to be immune privileged and allows xenograft growth^48^. Human fetal cartilage slices were grown in rabbit knee defects for 6 months without rejection or pannus formation^49^. Human amniotic mesenchymal stem cells were grown in rabbit knee joints for 8 weeks without obvious inflammatory responses^50^. Human ASCs were able to repair rabbit osteochondral defects and pig cartilage defects without immune reactions^51,52^. Human ASCs were able to regenerate cartilage in guinea pig knees^53^. Our present study shows that human ASCs-derived cartilage grew in rabbit and monkey knees without obvious host inflammatory responses for 6–24 months.

The cartilage repair outcomes were assessed both macroscopically and histologically. A previous study found that spontaneous cartilage repairs in older animals showed signs of degeneration at 48 weeks^54^. In the present study, we observed animals for up to 24 months. In the rabbits, macroscopic scores did not decrease from 6 months to 24 months (Supplementary Fig. 1). However, we did notice that the histological scores were slightly lower at 24 months than at 6 or 12 months, although the differences were not statistically significant (Supplementary Fig. 2). Yet, the DCX-eGFP pellets group still showed a significantly better histological score than the eGFP pellets group or the fibrin group in the rabbits at 24 months after surgery. Furthermore, the results from the monkeys at 24 months after surgery also showed that the DCX-eGFP pellets group had significantly better histological score than the eGFP pellets group or the fibrin group. These findings indicate that the cartilage tissues with DCX expression may sustain better quality of cartilage repair than the cartilage tissues without DCX expression.

Our findings showed that the neocartilage in the cartilage defects contained both hASCs-derived chondrocytes and host-derived chondrocytes as the chondrocytes came from both human and non-human origins. We speculate that the implanted hASC-derived chondrocytes might secrete some factors such as GDF5 to induce local in-migration and subsequent chondrocyte differentiation of host bone marrow mesenchymal stem cells. We found that the numbers of host-derived cells were not different between the DCX-eGFP pellets group and the eGFP pellets group, but the cells were counted based on the existing neocartilages. Given that the amount of neocartilages was significantly more in the DCX-eGFP pellets group than the eGFP pellets group, we could not rule out the possibility that DCX-induced GDF5 might increase egress of host bone marrow stem cells. Paracrine factors produced by the stem cells may provide stimulatory signals to the host cells. Saris and co-workers implanted a mixture of mesenchymal stem cells and recycled autologous chondrons (i.e., chondrocytes in their own territorial matrix) into goat chondral defects^55^ and human chondral defects in clinical trials^56–58^. They observed significantly better repair outcomes in the implant group compared to the microfracture group in the preclinical goat study and found long-lasting benefits in human studies with 12–60 months follow-up. Interestingly, biopsy of human neocartilages at 12 months after surgery did not find DNAs of allogeneic mesenchymal stem cells, suggesting that the allogeneic mesenchymal stem cells disappeared after providing stimulatory signals to the autologous chondrons via paracrine effects^56,57^. In our study, we detected exogenous hASCs-derived chondrocytes in the neocartilages at 24 months after surgery. It is unknown if the differentiated hASC-derived chondrocytes survive longer than undifferentiated stem cells, but future studies shall examine longer time period to clarify this. We found that DCX and GDF5 were co-expressed in the chondrocytes of the neocartilage. We showed both that DCX expression induced GDF5 expression and that GDF5 treatment enhanced DCX expression. Consistent with these observations, a previous study had shown that GDF5 induced DCX expression in mouse embryonic stem cells^14^. These findings suggest that there is a positive feedback loop between DCX and GDF5. In the embryonic joint development, DCX and GDF5 are co-expressed in the joint interzone cells during articular chondrocyte differentiation^12,37^. Expression of DCX in hASCs may increase GDF5 expression to promote articular chondrocyte differentiation. GDF5 has been shown to induce chondrocyte differentiation in limb mesenchymal cells^59^. GDF5-conjugated scaffolds with bone marrow stem cells have been shown to produce better repair than the scaffolds without GDF5 in the rabbit knee cartilage defect models^60^.

The findings from the present study imply that hASCs with DCX expression can be engineered to form cartilage tissues in vitro and used in articular cartilage repair in vivo, with potentially better repair outcomes than the cartilage tissues engineered without DCX expression. The materials used to fill the cartilage defects include cartilage tissues and hASCs with DCX expression, recombinant BMP7, and fibrin, which we collectively named as doublecortin-induced tissue engineered cartilage (DITEC). This DITEC technique has five unique features: (1) the sources of hASCs are abundant as about 280,000 liposuction procedures are done annually in the US^61^, thereby providing an abundant source of adipose tissues for hASCs isolation; (2) the chondrogenic media are chemically defined, which guarantees consistency in product quality and safety controls; (3) the 96-well-plate pellet culture system allows automation, which makes industrial-scale production possible; (4) the cartilage pellets and hASCs may be cryopreserved and stored as shelf-ready packages and delivered to the surgery room for on-site thawing, recovery, and use; and (5) hASCs may be used as autologous or allogenous. These unique features make it feasible to develop the DITEC technique into clinic use. The pellets are about 1 mm in size, which are similar to the particulated articular cartilage (also called minced cartilage or cartilage chips) as currently employed in the Cartilage Autograft Implantation System (CAIS, Depuy Mitek, Raynham, MA, USA) and DeNovo Natural Tissue (DeNovo NT, ISTO, St. Louis, MO, USA)^62^. Particulated articular cartilage has been tested in a variety of preclinical and clinical studies^62^, in which the small cartilage pieces are glued into the cartilage defects with a scaffold or fibrin, similar to the technical procedures employed in the present study. DeNovo NT uses fresh particulated juvenile (under the age of 13) articular cartilage from human donors and has been used to treat over 8700 patients from 2007 to 2015 with promising cartilage repair outcomes^62^. The limitation of DeNovo NT is its dependence on fresh cadaveric cartilage. This contrasts with the DITEC technique which uses tissue engineering to produce cartilage tissues from hASCs obtained from healthy living donors.

The limitation of the present study is that the cartilage defect repaired is quite small (about 0.1 cm^2^). Although the present study is successful as a proof-of-principle experiment, the DITEC technique will need to be tested in repair of large cartilage defects (e.g., 1 cm^2^ in size representing approximately 60% of the surface area of the rabbit knee femoral groove), in order to display feasibility for clinical translation to human cartilage repair. Another limitation is the use of lentiviruses to express DCX, which may present a safety concern. Although the lentiviruses are replication-incompetent, it would be advantageous to use alternative approaches such as adenovirus-associated virus or a combination of recombinant proteins (e.g., GDF5) to drive DCX expression in hASCs. The third limitation is the need for additional mechanical testing data. Although our original plan was to cut the harvested cartilage tissues into two halves, one for histology and one for mechanical testing. Due to the small size of the neocartilage, this proved to be unpractical. As a result, the majority of the samples were dedicated to the more informative histological evaluations rather than destructive mechanical testing. Consequently, we only used three rabbits each at 12 and 24 months after surgery to perform a pilot mechanical testing study, which is statistically underpowered. The fourth limitation is that the mechanism by which DCX improved the repair is unknown. We showed that DCX expression in hASCs increased expression of GDF5, matrilin 2, and collagen 2 in the pellets in vitro, GDF5/DCX double-positive chondrocytes were increased in the neocartilages of the DCX-eGFP group compared to the eGFP group, and recombinant GDF5 treatment increased DCX expression in the pellets in vitro. These results imply that DCX and GDF5 may regulate each other’s expression in a positive feedback loop, which may impact on the host-derived bone marrow stem cells through secreted GDF5. But the molecular mechanisms remain to be revealed. Nevertheless, the results from the present study encourage future studies to test the DITEC technique in repair of large cartilage defects in the preclinical animal models.

## Methods

### Study design

The purpose of this study was to test a hypothesis that DCX expression promotes differentiation of hASCs into articular chondrocytes and tissue engineered cartilage tissues with DCX expression may provide better cartilage repair outcomes than the ones without DCX expression. Human ASCs were transduced with lentiviruses expressing DCX-eGFP or eGFP (as control). Cartilage tissues were engineered using a 96-well pellet culture system. Critical size (3.5 mm diameter) cartilage defects were made in the femoral trochlear grooves of 42 rabbits and 12 rhesus macaques. DCX-eGFP- and eGFP-expressing cartilage tissues (pellets) were randomly assigned to fill in the distal defects, with the proximal defects filled with fibrin as untreated control. Animals and the defects were coded with letters and numbers, and the investigators responsible for surgeries and outcome assessment were blinded to the treatment or control groups. Group size was determined by power analysis using the effect size from previous studies^63–65^. No surviving animals were excluded from analysis and no outliers were excluded. Animals died prior to the endpoints were excluded from analysis. The number of biologic replicates is specified in the figure legends. All animal studies were approved by the Animal Care and Use Committee of Tulane University, which was in compliance with the U.S. Department of Health and Human Services Guide for the Care and Use of Laboratory Animals and the regulations of Department of Agriculture. The use of recombinant DNA was in compliance with the U.S. NIH Guidelines.

### Cells and DCX transduction

hASCs (passage zero) and stromal medium for stem cell culture were provided by LaCell, LLC (New Orleans, LA) through a grant funded subcontract. Four female donors (39, 41, 48, and 48 years old) were used and the hASCs from the four donors were tested to have equal chondrogenesis potential. The hASCs were isolated from lipoaspirate tissues gathered from elective surgical procedures and were characterized for immunophenotypes (CD29^+^CD34^+^CD73^+^CD90^+^CD105^+^CD44^low^CD45^low^) and differentiation properties (adipogenesis and osteogenesis)^26^. Briefly, the lipoaspirate tissue was extensively washed with warm phosphate-buffered saline (PBS) to remove erythrocytes and then digested in PBS supplemented with 0.1% Collagenase of Type I (Worthington Biochemical Corporation), 1% bovine serum albumin, and 2 mM CaCl_2_ for 1 h at 37 °C. Following room temperature centrifugation at 300 × *g* and resuspension in stromal medium [Dulbecco’s modified Eagle’s medium (DMEM)/Hams F-12 medium supplemented with 10% fetal bovine serum (Hyclone) and 1% antibiotic/antimycotic], the stromal vascular pellet was plated at a density of 35 ml of lipoaspirate digest per T175 flask (0.2 ml/cm^2^). After 24 h of incubation at 37 °C with 5% CO_2_, the adherent cells were washed with warm PBS and maintained in stromal medium until 80–90% confluent. The adherent population was harvested and frozen as passage zero stock^26^. Frozen hASCs were recovered for growth and then transduced with replication-incompetent lentiviruses (HRST-eGFP and HRST-DCX-GP-eGFP) as previously described^23^. Forty-eight hours later, transduction was verified under a fluorescent microscope and approximately 45% of hASCs were eGFP positive in both groups. The cells were not purified by flow cytometry cell sorting because the sorting procedure caused deaths of most of the cells. DCX expression was confirmed by western blot analysis as described^23^. Original blot images were scanned as color images using Odyssey^®^ Imaging System (software Image Studio version 3.1, LI-COR Biosciences, Lincohn, NE, USA). The images were inverted and changed to grayscale and then processed using Photoshop CC 2017 (Adobe, San Jose, CA, USA). Image brightness was adjusted linearly and equally across the entire image; contrast was not adjusted. Representative blot images were cropped and presented, while unprocessed original images were presented in Supplementary Information. All blots were derived from the same experiment and were processed in parallel.

### Ninety-six-well pellet culture system

Lentiviruses-transduced hASCs were plated in 96-well plates with “V”-shaped bottoms (50,000 cells per well in 0.3 ml chondrogenic medium). The plates were centrifuged at 453 × g for 5 min to form pellets. The chondrogenic medium contains DMEM supplemented with 10 ng/ml bone morphogenetic protein 7 (BMP7, R&D Systems, Minneapolis, MN), 2.5 ng/ml transforming growth factor β1 (TGFβ1, Thermo Fisher Scientific, Waltham, MA), 2.5 μg/ml human recombinant insulin, 2.5 μg/ml human transferrin, 2.5 ng/ml selenous acid, 2.1 μg/ml linoleic acid (BD Biosciences, San Jose, CA), 50 μg/ml 2-phospho-l-ascorbic acid trisodium salt (Sigma-Aldrich, St. Louis, MO), 1 mM sodium pyruvate (Invitrogen, Carlsbad, CA), 100 nM dexamethasone (Sigma-Aldrich), and 0.1% human serum albumin (Sigma-Aldrich). The pellets were cultured at 37 °C in a humidified incubator with 5% CO_2_ and 5% O_2_ (hypoxia). For the purpose of comparison, some pellets were cultured under normoxia (21% O_2_), which were not used in animal studies. The medium was changed every 2 days and cartilage tissues (named as eGFP pellets or DCX-eGFP pellets) were formed with a size of approximately 1 mm in diameter after 14 days of chondrogenic differentiation. The pellets were assessed for cartilage gene expression using real-time quantitative reverse transcription PCR (qRT-PCR) and IHC staining as described^23^. The culture media were confirmed to contain no bacteria/yeast/lentivirus growth and displayed endotoxin levels <0.06 endotoxin units/ml.

### Surgical implantation of the pellets

Forty-two New Zealand White rabbits (male:female = 1:1, retired breeders with estimated ages of about 1.5 years) were purchased from Charles River Laboratories (Wilmington, MA). Twelve rhesus macaques (male:female = 1:1, average age 7.24 ± 1.37 years) were obtained from and housed at Tulane National Primate Research Center (Covington, LA). Animals had not been treated with chondrogenic medium before implantation surgery. Animals were anesthetized with ketamine/xylazine (for rabbits) or ketamine/isoflurane (for monkeys). A single intravenous dose of Cefazolin antibiotics (20 mg/kg) was given prior to surgery. Analgesics (Buprenorphine for rabbits and Buprenorphine/Carprofen for monkeys) were given before and after surgery for pain relief. Both knees were shaved and scrubbed with alternating betadine and 70% ethanol. A 4-cm medial parapatellar skin incision was made in each knee, exposing the femoral trochlear groove. We used a sharp-tipped 2.8-mm drill bit to manually make a 2-mm hole on the trochlear cartilage to mark the localizations of the cartilage defects; then, we used a flat-tipped 3.2-mm drill bit (with a plastic stopper to stop drilling when the drill bit went 5-mm into the cartilage and subchondral bone) and an electric drill to make a defect of 3.5-mm in diameter and 5-mm in depth. The same critical size (3.5-mm in diameter) was used for the rabbit and monkey studies because the articular surface areas of femoral trochlear grooves were very close in sizes in both species. The procedures were repeated to make a second defect 5 mm away from the first one. Similarly, two defects were made in the other knee. Each knee was randomized to implant eGFP pellets or DCX-eGFP pellets in the distal defects, while the proximal defects were filled with fibrin sealant (TISSEEL, Baxter Health Corp, Westlake Village, CA). The distal defect was filled with 40 pellets mixed with 10 ng/ml BMP7 and one million lentiviruses (eGFP or DCX-eGFP)-transduced hASCs in TISSEEL. The rationale to mix the pellets with hASCs was that there were void spaces between the pellets and hASCs might fill in the spaces and differentiate into articular chondrocytes in the in vivo environment, while the pellets might provide instant mechanical support. Then, the joint capsule was closed with 5-0 or 3-0 absorbable sutures and the skin incision was closed with 5-0 or 3-0 absorbable sutures intradermally. After recovery from anesthesia, animals were returned to single housing cages and observed daily. Rabbits were weighed weekly. Monkeys were anesthetized for physical examination, blood counts, and blood chemistry every 3 months. All animals were followed up to their predetermined endpoints and most of them were healthy after surgery except three animals that were dead or euthanized due to illnesses (Supplementary Table 6). Animals were euthanized at the predetermined endpoints of 2 weeks, 6 months, 12 months, and 24 months after surgery for the rabbits and 24 months after surgery for the monkeys (Supplementary Table 7).

### Necropsy of animals

Necropsy of the euthanized rabbits was performed by the researchers. Knee joints and major organs (brain, heart, lungs, liver, kidneys, spleen, and intestines) were examined visually. Necropsy of the rabbits died prior to the endpoints was performed by the veterinarian who did visual examination and histological examination of the suspected organs. Necropsy of the monkeys was performed by the pathologists at Tulane National Primate Research Center, who examined the major organs (brain, heart, lungs, liver, kidneys, spleen, and intestines) visually and histologically and provided a necropsy report on each monkey.

### Evaluation of the cartilage repair outcomes

Cartilage repair outcomes were evaluated in a blinded manner in which the evaluator was blinded to the grouping of the repaired cartilage defects. Macroscopic evaluation followed the International Cartilage Repair Society (ICRS) assessment scales (Supplementary Table 1)^30–32^. Histological evaluation was based on tissue sections stained with Safranin O and immunohistochemical staining of collagen 1 and collagen 2 according to the modified ICRS visual histological assessment criteria (Supplementary Table 3)^33,34^.

### Cartilage tissue processing and staining

Femoral groove cartilage and subchondral bone were harvested en bloc, fixed in 4% paraformaldehyde, and kept in 70% ethanol. The samples were demineralized for 18–21 days in the Immunocal reagent (Decal Chemical Corp., Tallman, NY). Then, the samples were embedded in paraffin and cut into 3-µm-thick tissue sections. Tissue sections were stained with Safranin O. Briefly, tissue sections were stained with hematoxylin solution for 8 min and washed in running tap water for 5 min; then, tissue sections were stained with 0.25% Light Green SF Yellowish (Thermo Fisher Scientific, Waltham, MA) for 1 min and rinsed quickly with 1% acetic acid solution for 15 s; finally, tissue sections were stained with 1% Safranin O solution (Thermo Fisher Scientific, Waltham, MA) for 30 min. Alcian blue staining was performed as described^66^. Immunohistochemical staining and immunofluorescent double staining of the pellets and cartilage tissues were performed as described previously^11,12^. The antibodies used are listed in Supplementary Table 8. All photomicrographs were captured using the EVOS^®^ FL Auto Imaging System (software EVOS, Life Technologies, Carlsbad, CA, USA) that was equipped with ×4, ×10, ×20, and ×40 objective lenses, CMOS color camera, and CCD monochrome camera, and that was capable to perform automated bright-field and multichannel fluorescence imaging using built-in filters and detectors. The images were processed using PowerPoint 2013 and Photoshop CC 2017, without any gamma changes or deconvolution. The resolution at which an image was acquired was indicated in each figure and figure legend.

### Mechanical testing

The cartilage underwent step-wise stress-relaxation experiments using unconfined compression mechanical testing (BioDynamic 5170 System, displacement resolution = 1 µm, load cell resolution = 0.006 N; TA Intruments-Electroforce, New Castle, DE)^67–69^. We used a 3.5-mm diameter biopsy punch to collect intact full thickness cartilage disks and subchondral bone from eight rabbits^70^. The punches were made through the cartilage defects that were repaired with TISSEEL (fibrin sealant), eGFP pellets, and DCX-eGFP pellets, as well as adjacent host cartilage (used as normal cartilage control). A stereomicroscope (Olympus SZX12; Olympus Corporation, Tokyo, Japan) equipped with a digital camera and CaptaVision software (Excelis MPX 16C; Accu-Scope, Commack, NY, USA) was used to capture images of the cartilage. ImageJ (NIH; Bethesda, MD) was used to quantify cartilage unloaded cross-sectional area and thickness. A digital caliper (CD-AX series, resolution: 0.01 mm; Mitutoyo Corporation, Sakado, Takatsu-ku, Kawasaki, Kanagawa, Japan) was used to measure the unloaded thickness of the intact bone and cartilage. The samples were placed with the bone side down on the bottom compression platen. Sandpaper on the top and bottom nonporous platens was used to secure the sample. The cartilage was submerged in Hanks balanced salt solution at room temperature (20 °C) throughout the mechanical test. A contact tare load of 0.04–0.08 N ensured good contact between the sample and platens^68^, followed by 10 min of equilibration ensuring a steady and constant load reading^67^. Sinusoidal loading at 2% strain over five cycles at 0.5 Hz preconditioned the cartilage, followed by 10 min of equilibration^67^. Stress-relaxation protocols were performed at 1 µm/s^68–70^ over five steps with each step 4% of the uncompressed cartilage thickness and 10 min of relaxation^71^. Stress was calculated by normalizing load to the unloaded cross-sectional area. The equilibrium modulus was calculated by taking the slope of the equilibrium stress versus strain data^69,72^. All samples from one rabbit at 6 months and one rabbit at 12 months were used for pilot studies to determine sample preparation (i.e., intact bone versus no bone) for mechanical testing, thus being excluded from data analysis. Samples where the bone was not intact with the cartilage were excluded from the mechanical test, hence one left TISSEEL (fibrin sealant) at 12 months, one left TISSEEL at 24 months, and one right TISSEEL at 24-months were not included in the data analysis. At 12 months, this resulted in the distribution of six samples from the normal group (three left and three right), three samples from DCX-eGFP pellets group, three samples from the eGFP pellets group, and five samples from TISSEEL group (two left and three right). At 24 months, this resulted in the distribution of six samples from the normal group (three left and three right), three samples from the DCX-eGFP pellets group, three samples from the eGFP pellets group, and four samples from the TISSEEL group (two left and two right). A one-way analysis of variance (ANOVA) evaluated differences in the equilibrium modulus between the normal cartilage group, DCX-eGFP pellets group, eGFP pellets group, and TISSEEL group. Unpaired *t*-test evaluated difference in the equilibrium modulus between 12 and 24 months.

### Statistical analysis

All measurements were taken from distinct samples and sample size (*n*) for each experimental group was given in the figure legends. Prism 8 software (GraphPad Software, San Diego, CA) was used for statistical analyses. The data are reported as mean ± standard deviation (SD) or as specified. All statistical analyses were performed with one-way analysis of variance (ANOVA), two-sided, unless otherwise noted. Kruskal–Wallis test was used to evaluate the overall macroscopic repair outcomes in Supplementary Table 2. Statistical significance was determined as *P* < 0.05.

### Reporting summary

Further information on research design is available in the Nature Research Reporting Summary linked to this article.

## Supplementary information

Supplementary Information

Reporting Summary

## Data Availability

All data associated with this study are present in the paper or the Supplementary Information. All relevant data are available from the authors.
