# Supplementary material for: Human adipose-derived stromal/stem cells expressing doublecortin improve cartilage repair in rabbits and monkeys
Source: NPJ Regen Med. 2021 Nov 30;6:82. doi: 10.1038/s41536-021-00192-6 (PMC8633050; doi:10.1038/s41536-021-00192-6)
Supplement: Supplementary file 1 — Supplementary Information [file 41536_2021_192_MOESM1_ESM.pdf]

Supplementary Information for

Human adipose-derived stromal/stem cells expressing doublecortin improve cartilage repair in rabbits and monkeys

Dongxia Ge, Michael J. O'Brien, Felix H. Savoie, Jeffrey M. Gimble, Xiying Wu, Margaret H. Gilbert, Gabrielle L. Clark-Patterson, Jason D. Schuster, Kristin S. Miller, Alun Wang, Leann Myers, Zongbing You*

*Corresponding author. Email: zyou@tulane.edu

The PDF file include:

Supplementary Figure 1. Macroscopic scores of repaired cartilage defects in the rabbits.

Supplementary Figure 2. Histological scores of repaired cartilage defects in the rabbits.

Supplementary Figure 3. Mechanical response of rabbit cartilage under unconfined compression testing.

Supplementary Figure 4. IHC of eGFP in rabbit cartilage tissues at 12 and 24 months after surgery.

Supplementary Figure 5. IHC of Ku80 and human nuclei/mitochondria in monkey cartilage tissues at 24 months after surgery.

Supplementary Figure 6. IHC of Ku80 and human nuclei/mitochondria in rabbit cartilage tissues at 24 months after surgery.

Supplementary Figure 7. Immunofluorescent double staining of GDF5 and DCX in rabbit cartilage tissues at 24 months after surgery and gene expression in *in-vitro* cultured cartilage pellets.

Supplementary Figure 8. IHC of type X collagen in the neocartilage tissues of rabbits and monkeys.

Supplementary Figure 9. IHC of CD45 in the neocartilage tissues of rabbits and monkeys.

Supplementary Figure 10. Number of host derived cells in the rabbit and monkey neocartilages at 24 months after surgery.

Supplementary Figure 11. Unprocessed original images of Western blots in Figure 1b.

Supplementary Table 1. International Cartilage Repair Society (ICRS) macroscopic evaluation of cartilage repair.

Supplementary Table 2. Overall macroscopic repair outcomes.

Supplementary Table 3. Modified International Cartilage Repair Society (ICRS) visual histological assessment criteria.

Supplementary Table 4. ICRS histological scores of each evaluation category.

Supplementary Table 5. Gender differences in ICRS scores between male and female animals.

Supplementary Table 6. Animal health and cause of mortality.

Supplementary Table 7. Animal numbers and grouping.

Supplementary Table 8. List of antibodies used.

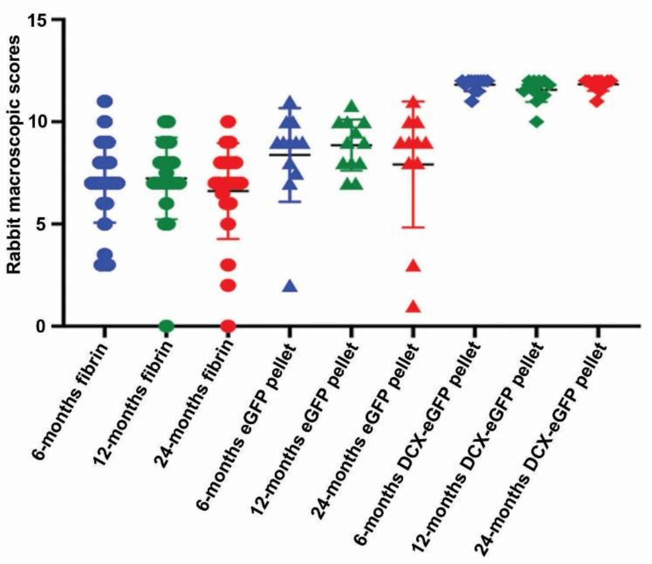

Supplementary Figure 1. Macroscopic scores of repaired cartilage defects in the rabbits. The differences within each treatment group (fibrin, eGFP pellets, and DCX-eGFP pellets) at different endpoints (6, 12, and 24 months after surgery) were not statistically significant.

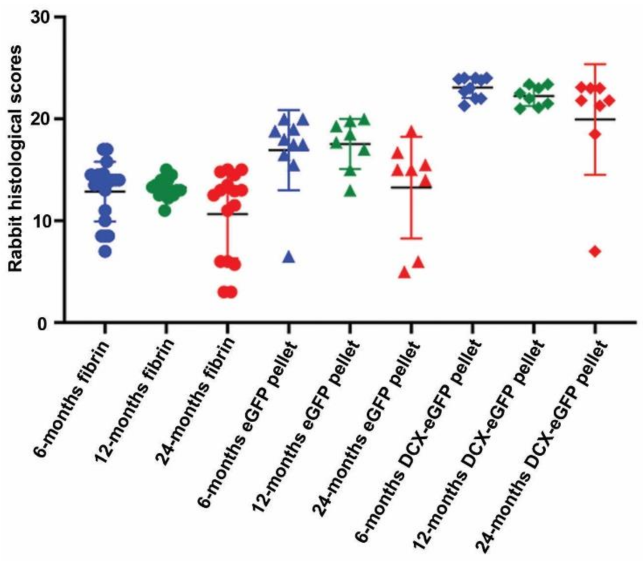

Supplementary Figure 2. Histological scores of repaired cartilage defects in the rabbits. The differences within each treatment group (fibrin, eGFP pellets, and DCX-eGFP pellets) at different endpoints (6, 12, and 24 months after surgery) were not statistically significant.

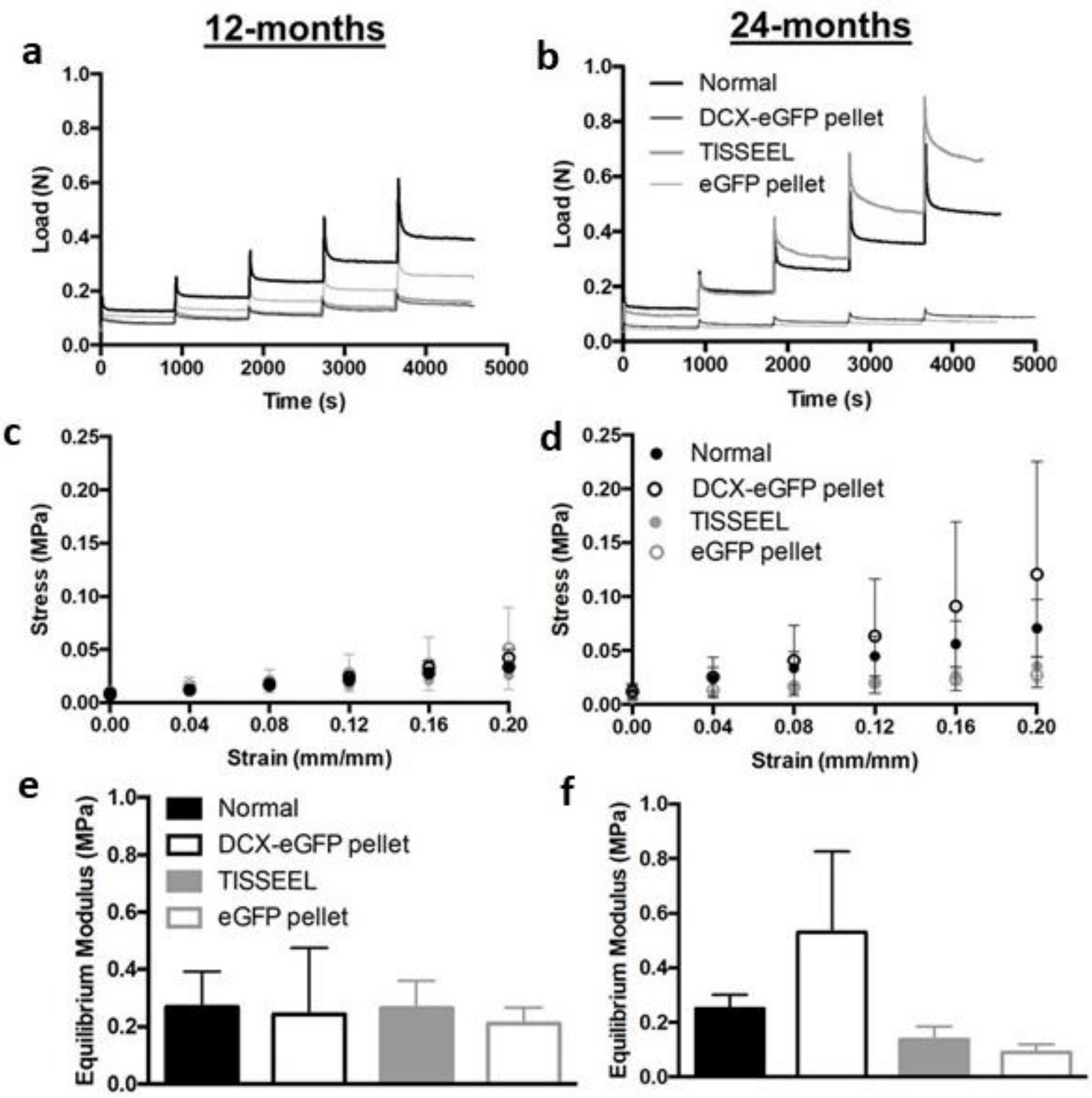

Supplementary Figure 3. Mechanical response of rabbit cartilage under unconfined compression testing. The repaired cartilage on a layer of subchondral bone was placed on the platform. The thickness of cartilage was measured. Load (force) was applied to the cartilage to calculate the strain (equals to the change of thickness) and stress (equals to the load/area). Representative load-relaxation responses are shown in **a** for 12-months samples and **b** for 24-months samples. Representative equilibrium modulus representing stress-strain responses are shown in **c** for 12-months samples and **d** for 24-months samples. Data of the average equilibrium modulus describing the stiffness of the material at equilibrium for **e** 12-months samples and **f** 24-months samples are presented. The equilibrium modulus was not statistically significantly different (*P*>0.05) among the cartilage types and between 12-months and 24-months points. Data are presented as mean ± standard error of the mean (error bars). Sample numbers are: **e** Normal n=6, DCX-eGFP pellets group n=3, TISSEEL group n=5, and eGFP pellets group n=3; **f** Normal n=6, DCX-eGFP pellets group n=3, TISSEEL group n=4, and eGFP pellets group n=3.

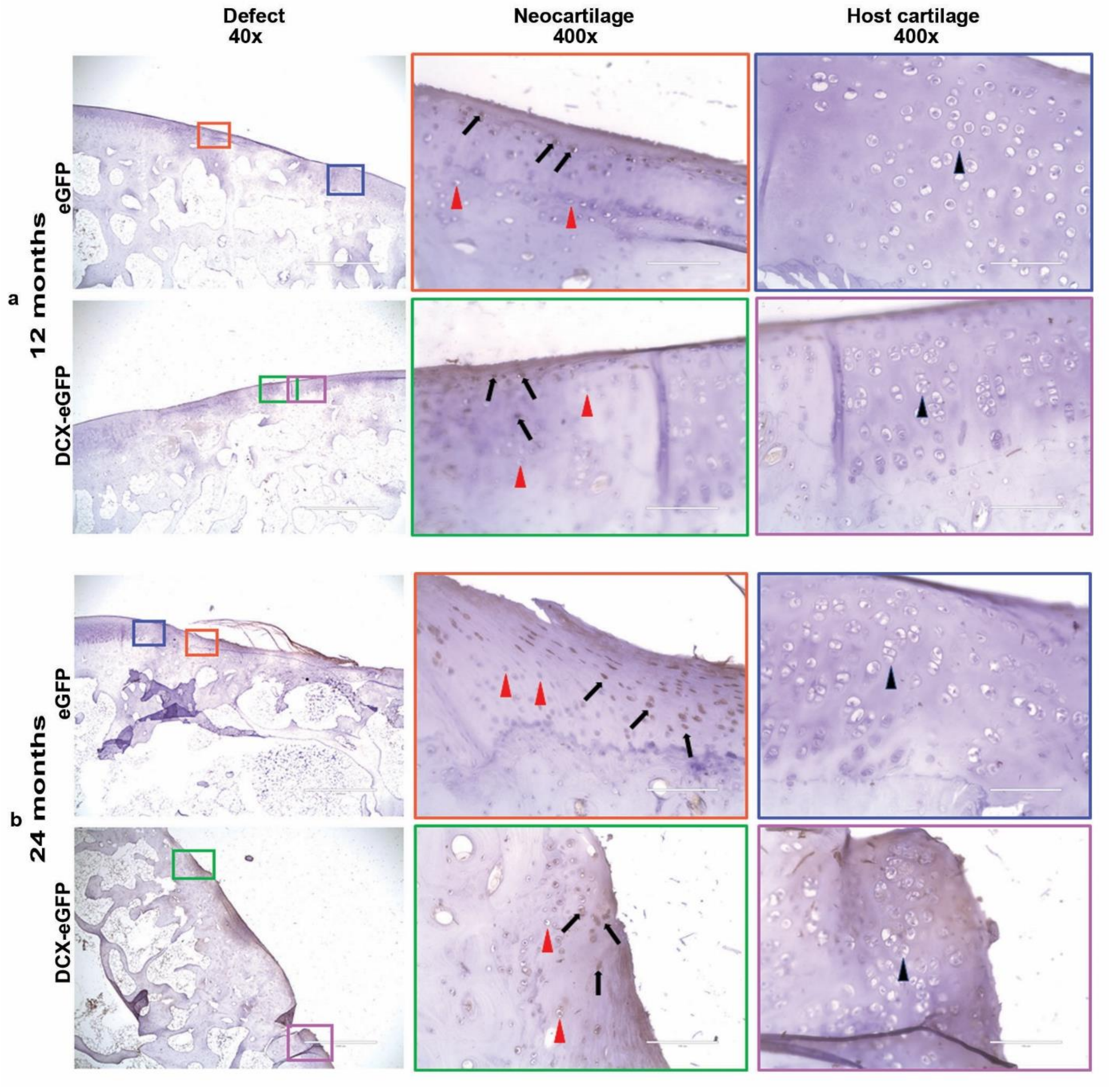

Supplementary Figure 4. IHC of eGFP in rabbit cartilage tissues at 12 and 24 months after surgery. a-b Representative photomicrographs of IHC staining of eGFP in the rabbit cartilage tissues at 12 and 24 months after surgery; color-coded regions of interest in the 40x field (scale bars = 1000 µm) were magnified under 400x (scale bars = 100 µm); arrows, eGFP-positive chondrocytes; red arrowheads, eGFP-negative chondrocytes in the neocartilage; black arrowheads, eGFP-negative chondrocytes in the host cartilage.

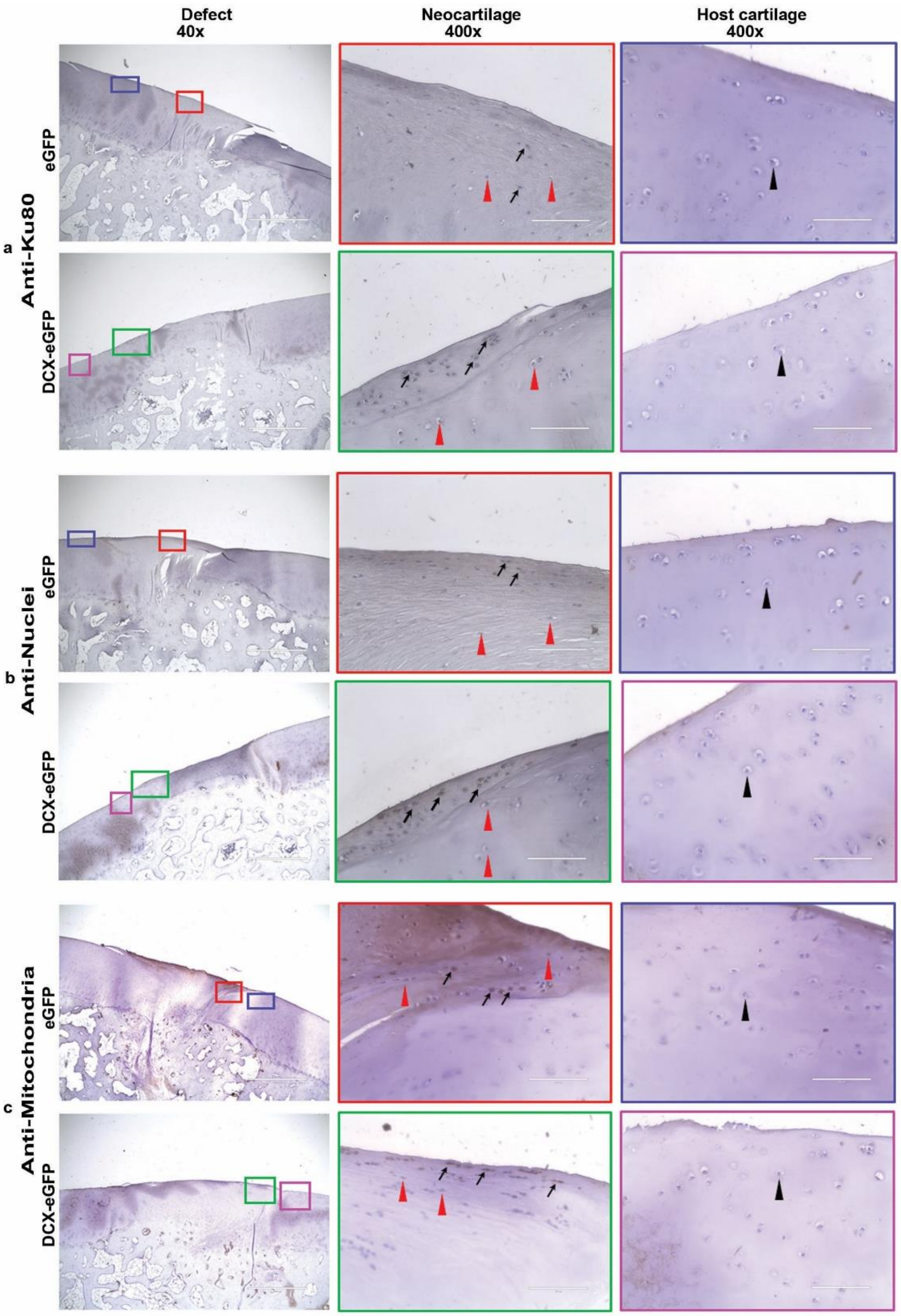

Supplementary Figure 5. IHC of Ku80 and human nuclei/mitochondria in monkey cartilage tissues at 24 months after surgery. a Representative photomicrographs of IHC staining of Ku80; color-coded regions of interest in the 40x field (scale bars = 1000 µm) were magnified under 400x (scale bars = 100 µm); arrows, Ku80-positive chondrocytes; red arrowheads, Ku80-negative chondrocytes in the neocartilage; black arrowheads, Ku80-negative chondrocytes in the host cartilage. **b** Representative photomicrographs of IHC staining of human nuclei; color-coded regions of interest in the 40x field (scale bars = 1000 µm) were magnified under 400x (scale bars = 100 µm); arrows, human nuclei-positive chondrocytes; red arrowheads, human nuclei-negative chondrocytes in the neocartilage; black arrowheads, human nuclei-negative chondrocytes in the host cartilage. **c** Representative photomicrographs of IHC staining of human mitochondria; color-coded regions of interest in the 40x field (scale bars = 1000 µm) were magnified under 400x (scale bars = 100 µm); arrows, human mitochondria-positive chondrocytes; red arrowheads, human mitochondria-negative chondrocytes in the neocartilage; black arrowheads, human mitochondria-negative chondrocytes in the host cartilage.

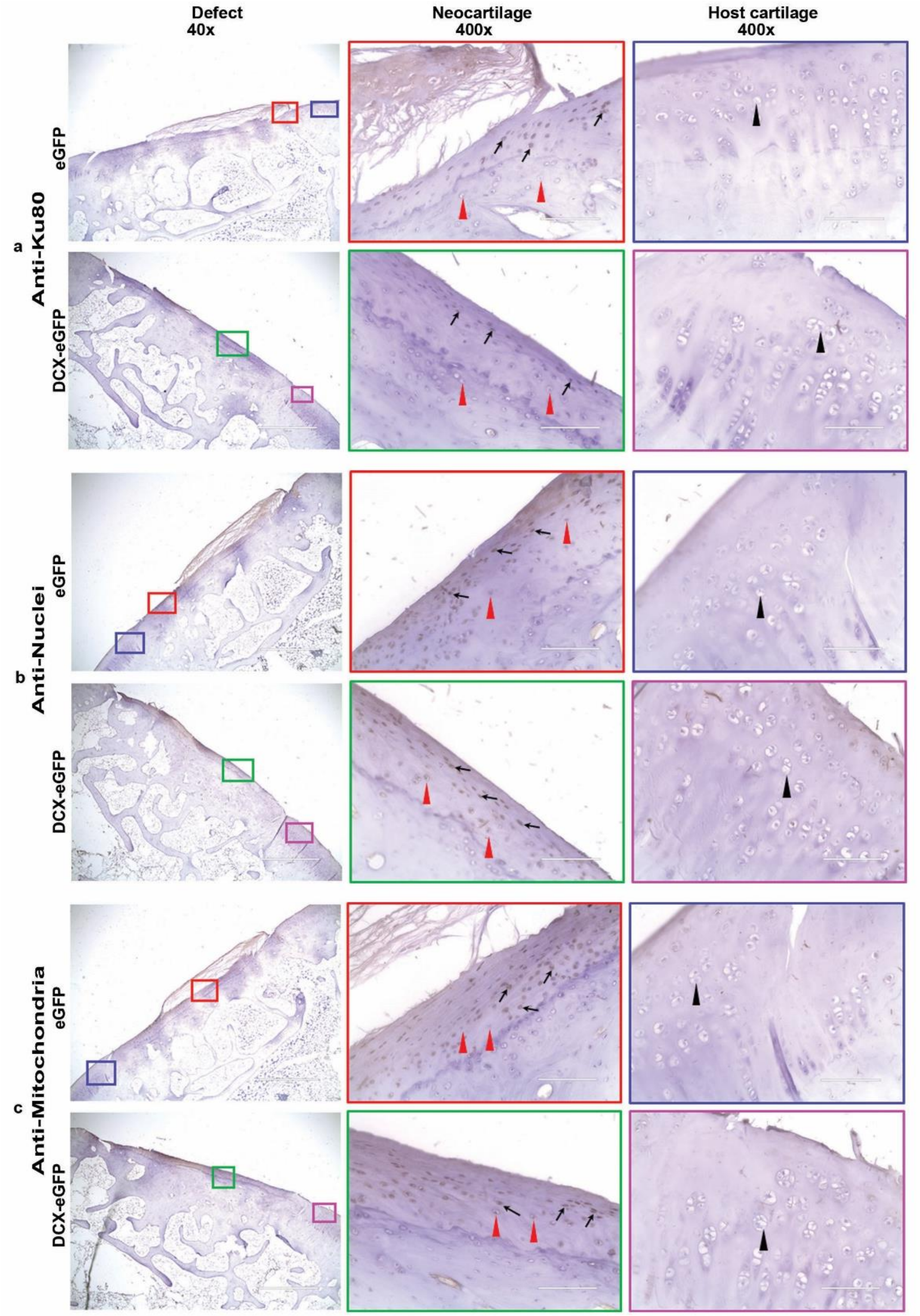

Supplementary Figure 6. IHC of Ku80 and human nuclei/mitochondria in rabbit cartilage tissues at 24 months after surgery. a Representative photomicrographs of IHC staining of Ku80; color-coded regions of interest in the 40x field (scale bars = 1000 µm) were magnified under 400x (scale bars = 100 µm); arrows, Ku80-positive chondrocytes; red arrowheads, Ku80-negative chondrocytes in the neocartilage; black arrowheads, Ku80-negative chondrocytes in the host cartilage. **b** Representative photomicrographs of IHC staining of human nuclei; color-coded regions of interest in the 40x field (scale bars = 1000 µm) were magnified under 400x (scale bars = 100 µm); arrows, human nuclei-positive chondrocytes; red arrowheads, human nuclei-negative chondrocytes in the neocartilage; black arrowheads, human nuclei-negative chondrocytes in the host cartilage. **c** Representative photomicrographs of IHC staining of human mitochondria; color-coded regions of interest in the 40x field (scale bars = 1000 µm) were magnified under 400x (scale bars = 100 µm); arrows, human mitochondria-positive chondrocytes; red arrowheads, human mitochondria-negative chondrocytes in the neocartilage; black arrowheads, human mitochondria-negative chondrocytes in the host cartilage.

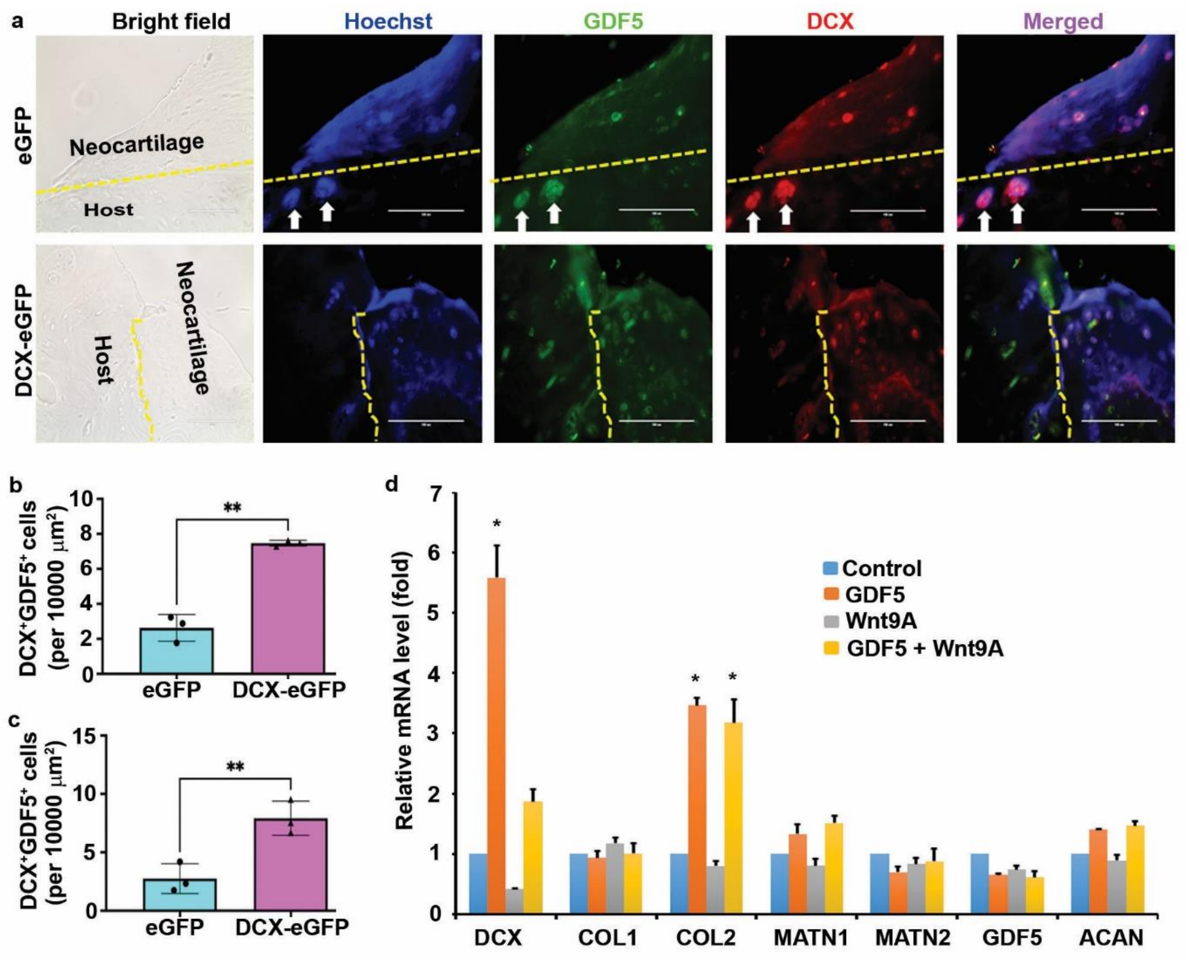

Supplementary Figure 7. Immunofluorescent double staining of GDF5 and DCX in rabbit cartilage tissues at 24 months after surgery and gene expression in *in-vitro* cultured cartilage pellets. a Representative photomicrographs of immunofluorescent double staining of GDF5 and DCX in rabbit cartilage tissues at 24 months after surgery; dotted lines outline the boundaries between the neocartilage and host cartilage; many GDF5/DCX double-positive chondrocytes are shown in the neocartilage of DCX-eGFP pellets group and isolated GDF5/DCX double-positive chondrocytes are shown in the neocartialge of eGFP pellets group; arrows indicate clusters of GDF5/DCX double-positive chondrocytes at the boundary between the neocartilage and host cartilage, which might be clusters of proliferating host chondrocytes in an attempt to repair the cartilage defect; scale bars = 100 μm. **b** Quantification of DCX/GDF5 double-positive chondrocytes in the rabbit neocartilage; n=3 randomly selected animals; $**P<0.01$. **c** Quantification of DCX/GDF5 double-positive chondrocytes in the monkey neocartilage; n=3 randomly selected animals; $**P<0.01$. **d** qRT-PCR analysis of the mRNA levels of collagen 1 (*COL1*), collagen 2 (*COL2*), matrilin 1 (*MATN1*), matrilin 2 (*MATN2*), growth differentiation factor 5 (*GDF5*), and aggrecan (*ACAN*) in *in-vitro* cultured cartilage pellets; data represent mean ± standard deviation (error bars) of 10 pellets per group (n=10); $*P<0.05$, compared to the control group. Of note, recombinant Wnt9A was included in the analysis because Wnt9A, like GDF5 and DCX, was expressed in joint interzone during the process of chondrocyte differentiation, hence it was included in comparison with GDF5.

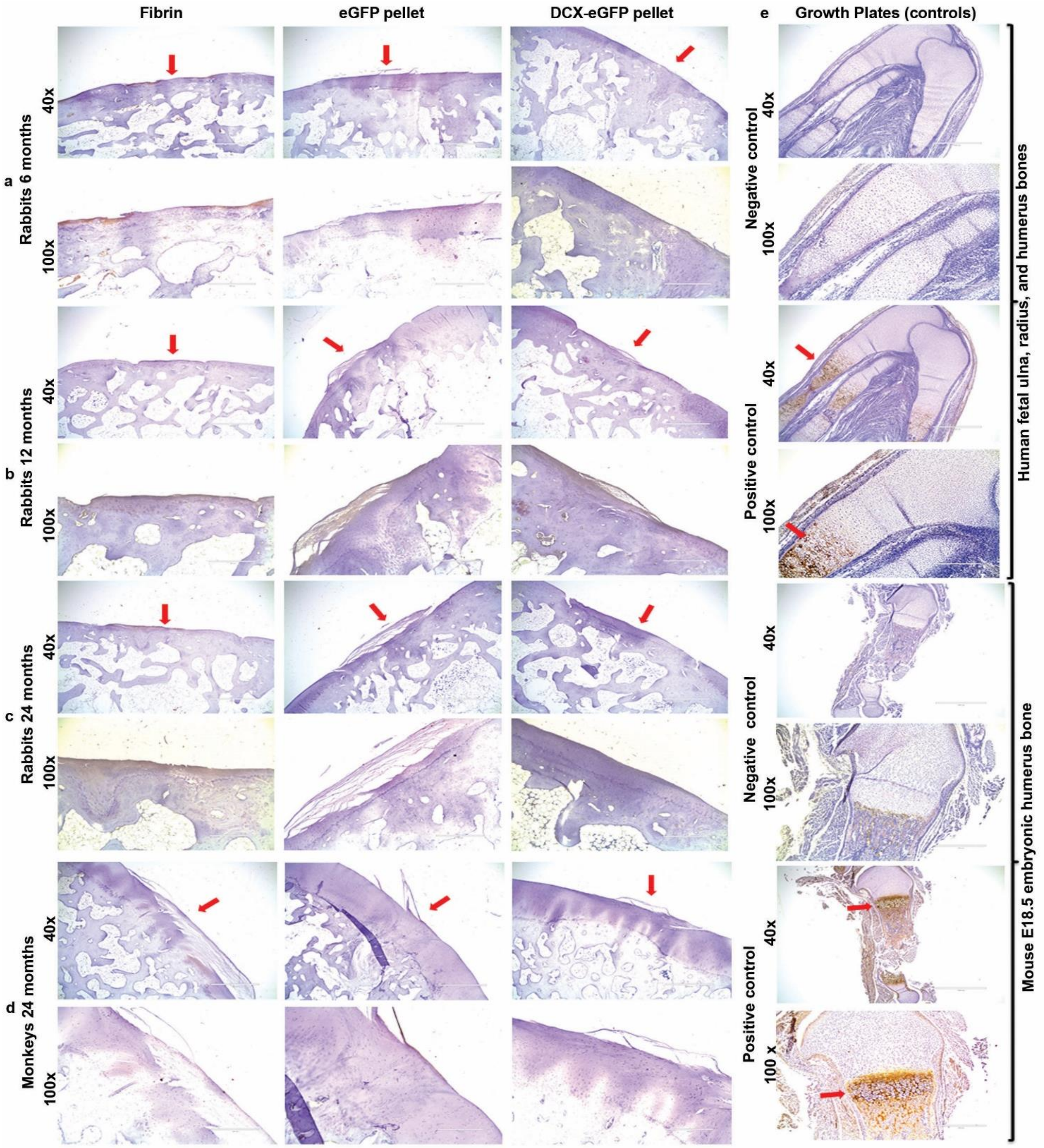

Supplementary Figure 8. IHC of type X collagen in the neocartilage tissues of rabbits and monkeys. a-d Representative photomicrographs of IHC staining of neocartilage tissues from 3 randomly selected animals in each group; arrows, repaired cartilage defects (neocartilage tissues). **e** Representative photomicrographs of IHC staining of the growth plates of human fetal ulna, radius, and humerus bones (archived and de-identified specimens obtained from our previous study) and mouse E18.5 embryonic humerus bone (archived specimens from our previous study), which served as negative and positive controls; negative control, phosphate-buffered saline replaced primary anti-collagen X antibodies; positive control, the growth plates were stained positive (arrows) for type X collagen. 40x, scale bars = 1000 µm; 400x, scale bars = 100 µm.

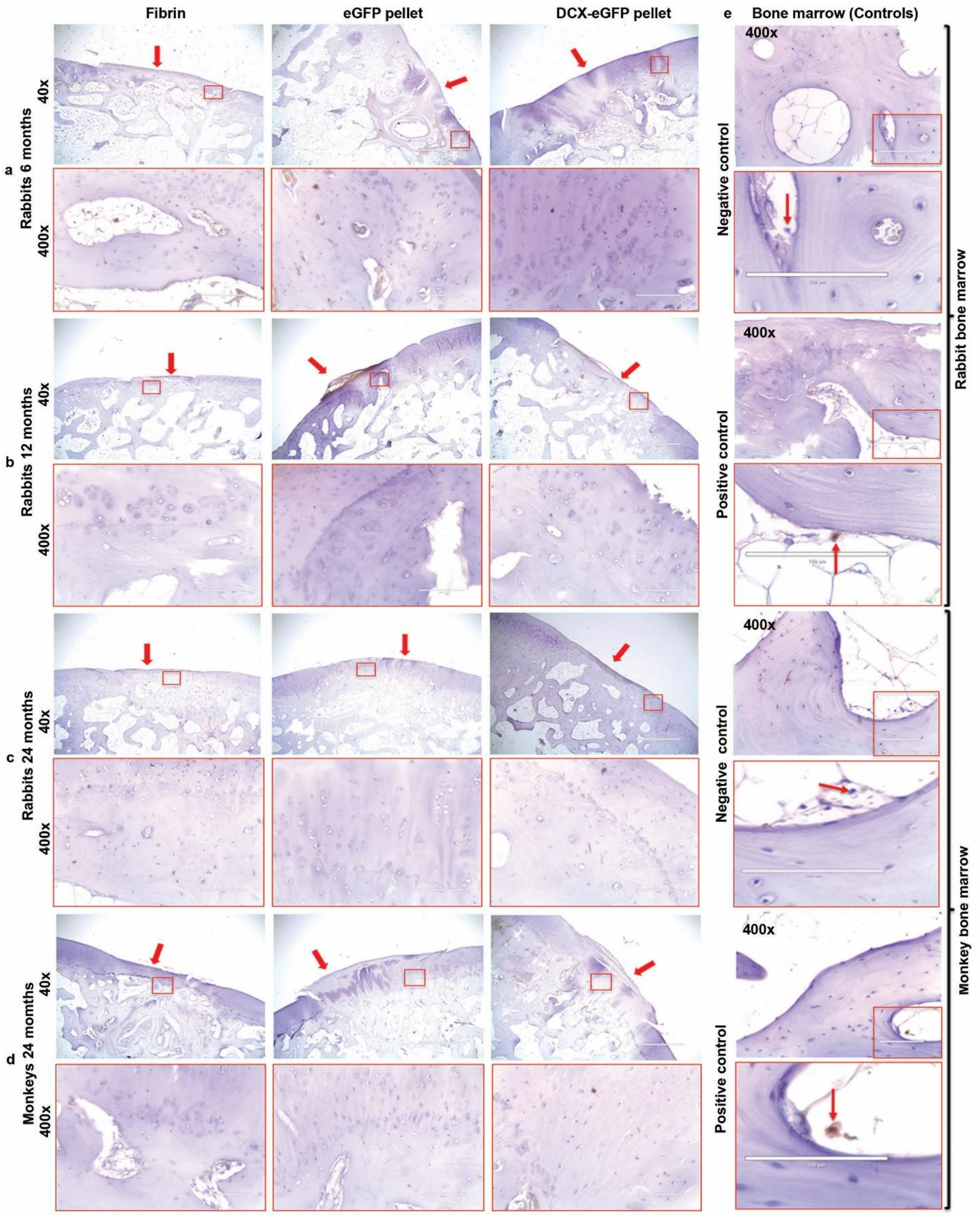

Supplementary Figure 9. IHC of CD45 in the neocartilage tissues of rabbits and monkeys. a-d Representative photomicrographs of IHC staining of neocartilage tissues from 3 randomly selected animals in each group; arrows, repaired cartilage defects (neocartilage tissues); selected regions in the 40x fields were magnified to 400x. **e** Representative photomicrographs of IHC staining of the rabbit and monkey bone marrow tissues, which served as negative and positive controls; negative control, phosphate-buffered saline replaced primary anti-CD45 antibodies; positive control, the leukocytes were stained positive for CD45; selected regions were expanded further to highlight the leukocytes (arrows). 40x, scale bars = 1000 µm; 400x, scale bars = 100 µm.

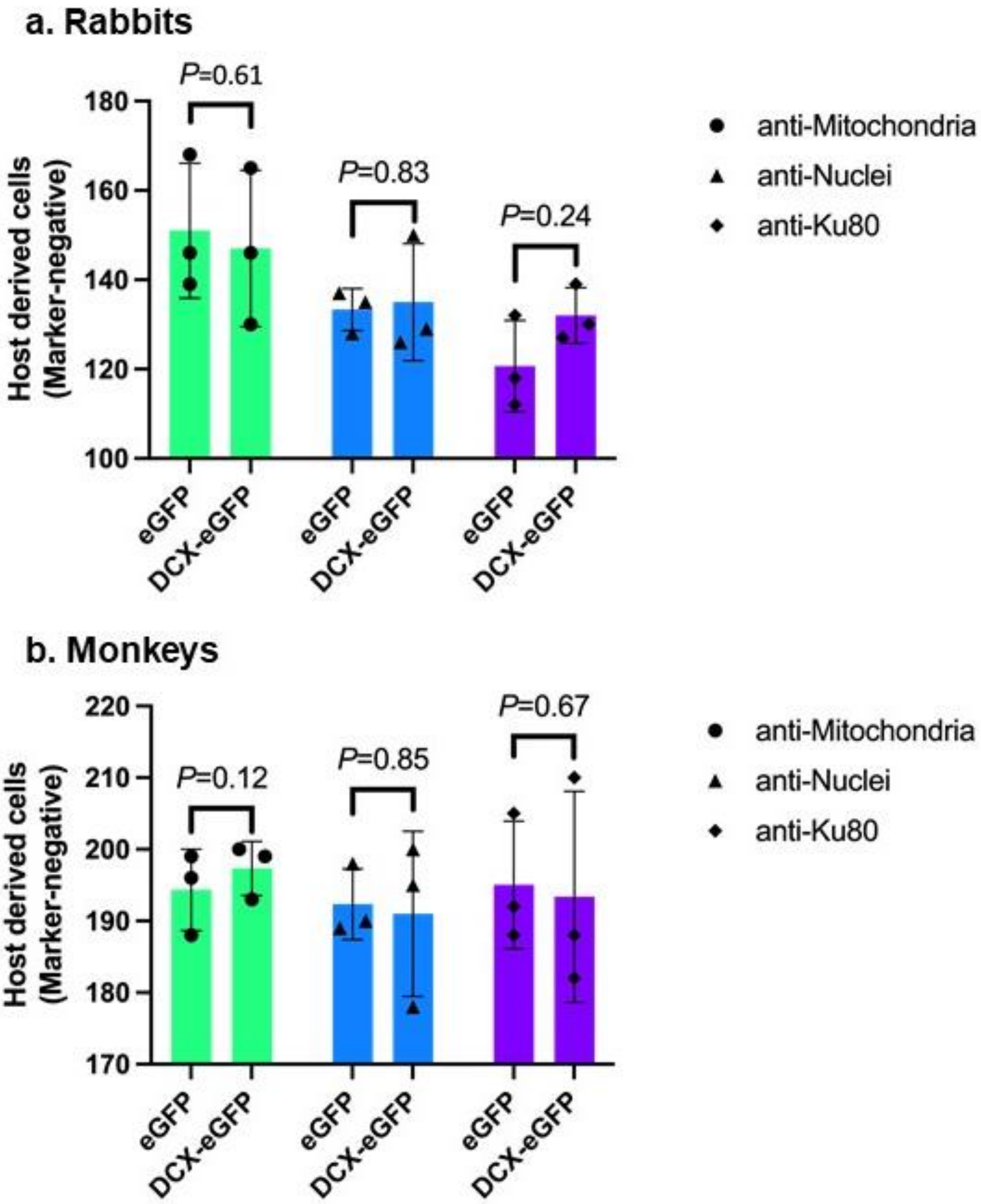

Supplementary Figure 10. Number of host derived cells in the rabbit and monkey neocartilages at 24 months after surgery. a-b Quantification of host derived cells (marker-negative) in the rabbit and monkey neocartilages per low-power field (x40 magnification); n=3 randomly selected animals; *P* values were obtained using Student's t test (two sided).

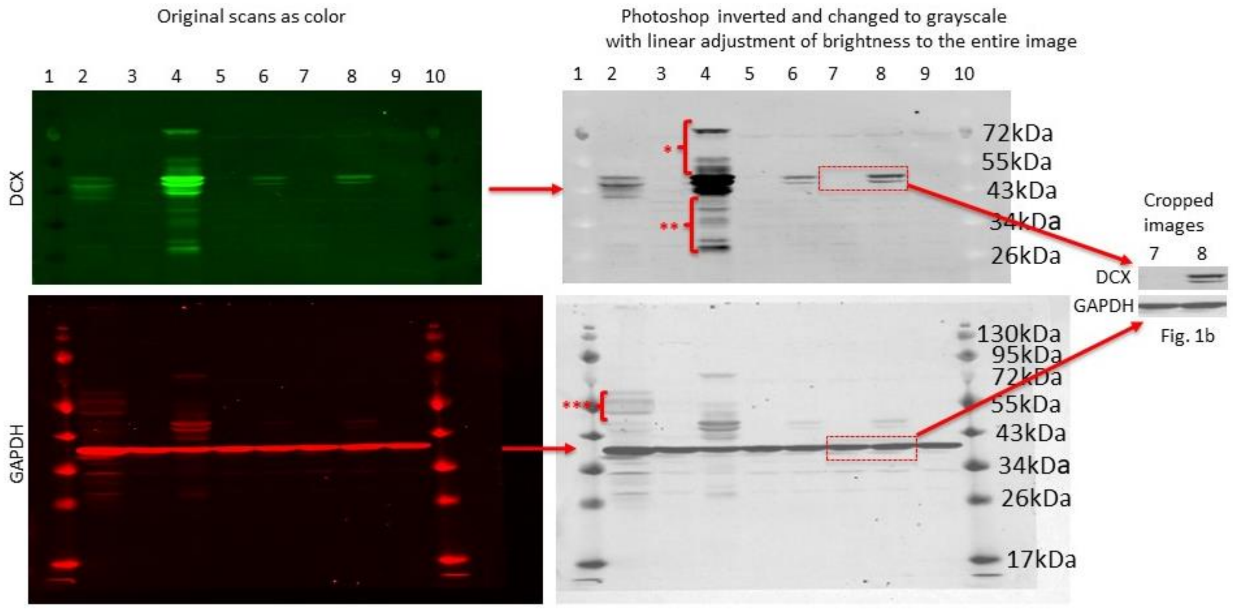

Supplementary Figure 11. Unprocessed original images of Western blots in Figure 1b. The samples were loaded in the following order as marked above the lanes:

1. Fisher BioReagents™ EZ-Run™ Prestained Rec Protein Ladder;
2. Mouse brain protein as positive control for DCX expression because immature neurons express DCX.
3. 293 cells transduced with eGFP lentiviruses (negative control for lane #4);
4. 293 cells transduced with DCX-eGFP lentiviruses (used to assess lentiviruses' infectiousness);
5. hASCs transduced with eGFP lentiviruses (control for lane #6, batch #1);
6. hASCs transduced with DCX-eGFP lentiviruses (batch #1);
7. hASCs transduced with eGFP lentiviruses (control for lane #8, batch #2);
8. hASCs transduced with DCX-eGFP lentiviruses (batch #2);
9. 293 cells without any transduction as negative control for DCX expression;
10. Fisher BioReagents™ EZ-Run™ Prestained Rec Protein Ladder; molecular weight is indicated.

The blot membrane was first probed for DCX and then for GAPDH as protein loading control. The original images were scanned as color images because the secondary antibodies were fluorescent. The original images were scanned using Odyssey® Imaging System (software Image Studio version 3.1, LI-COR Biosciences, Lincohn, NE, USA). The images were inverted and changed to grayscale using Photoshop CC 2017 (Adobe, San Jose, CA, USA). Image brightness was adjusted linearly and equally across the entire image; contrast was not adjusted. Of note, GAPDH image appears larger than DCX image because GAPDH image scan included some extra space outside of the blot membrane. Both images were scanned from the same membrane as evidenced by some reminiscent DCX signals on the GAPDH image. Representative lanes 7 and 8 were cropped to be presented in Figure 1b, though lanes 5 and 6 could also be chosen instead. *The bands with higher molecular weight might be some modified forms of DCX such as phosphorylation and ubiquitination; ** the bands with lower molecular weight might be some degraded forms of DCX due to overt overexpression of DCX in 293 cells. ***These bands might be mouse IgG heavy chains contained in the mouse brain tissues, which were recognized by anti-mouse secondary antibodies because anti-GAPDH primary antibody was of mouse origin, while anti-DCX primary antibody was of goat origin.

Supplementary Table 1. International Cartilage Repair Society (ICRS) macroscopic evaluation of cartilage repair.

Cartilage repair assessment scale	Score
Degree of defect repair	
In level with surrounding cartilage	4
75% repair of defect depth	3
50% repair of defect depth	2
25% repair of defect depth	1
0% repair of defect depth	0
Integration to border zone	
Complete integration with surrounding cartilage	4
Demarcating border <1 mm	3
¾ of graft integrated, ¼ with a notable border > 1 mm wide	2
½ of graft integrated with surrounding cartilage, ½ with a notable border > 1 mm	1
From no contact to ¼ of graft integrated with surrounding cartilage	0
Macroscopic appearance	
Intact smooth surface	4
Fibrillated surface	3
Small, scattered fissures or cracks	2
Several, small or few but large fissures	1
Total degeneration of grafted area	0
Overall repair assessment	
Grade I: normal	12
Grade II: nearly normal	11-8
Grade III: abnormal	7-4
Grade IV: severely abnormal	3-1

Supplementary Table 2. Overall macroscopic repair outcomes.

	Group	6 months						12 months						24 months					
		Defect	Grade				*P*	Defect	Grade				*P*	Defect	Grade				*P*
		#	I	II	III	IV		#	I	II	III	IV		#	I	II	III	IV	
Rabbits	Fibrin	24	0	9	12	3	0.000	24	0	10	13	1	0.0001	22	0	8	11	3	0.000
	eGFP	12	0	9	2	1		12	0	10	2	0		11	0	9	0	2	
	DCX-eGFP	12	8	4	0	0		12	4	8	0	0		11	7	4	0	0	
Monkeys	Fibrin													22	0	7	12	3	0.000
	eGFP													11	1	7	1	2	
	DCX-eGFP													11	8	3	0	0	

Supplementary Table 3. Modified International Cartilage Repair Society (ICRS) visual histological assessment criteria.

Category	Score
A The regularity of the surface	
Smooth/continuous	3
Irregularities/discontinuous	0
B Matrix morphology	
Hyaline	3
Mixture: hyaline/fibrocartilage	2
Fibrocartilage	1
Fibrous tissue	0
C Cell distribution	
Columnar	3
Mixed/columnar-clusters	2
Clusters	1
Individual cells/disorganized	0
D Cell population viability	
Predominantly viable	3
Partially viable	1
<10% viable	0
E Subchondral bone	
Normal	3
Increased remodeling	2
Bone necrosis/granulation tissue	1
Detached/fracture/callus at base	0
F Cartilage mineralization (calcified cartilage)	
Normal	3
Abnormal/inappropriate location	0
G Type I collagen staining of the matrix	
None	3
Slight	2
Moderate	1
Abundant	0
H Type II collagen staining of the matrix	
Abundant	3
Moderate	2
Slight	1
None	0
Total score	24

Supplementary Table 4. ICRS histological scores of each evaluation category.

Category	Rabbits												Monkeys			
	6-months (mean ± SD)				12-months (mean ± SD)				24-months (mean ± SD)				24-months (mean ± SD)			
	Fibrin	eGFP	DCX-eGFP	*P* value	Fibrin	eGFP	DCX-eGFP	*P* value	Fibrin	eGFP	DCX-eGFP	*P* value	Fibrin	eGFP	DCX-eGFP	*P* value
**A**. The regularity of the surface	1.43±0.86	1.90±0.88	2.83±0.33	0.0002	1.30±0.58	1.81±1.10	2.80±0.34	0.0001	0.71±0.72	1.19±0.96	2.48±0.71	<0.0001	1.14±0.86	1.82±0.81	2.69±0.45	<0.0001
**B**. Matrix morphology	0.86±0.57	1.60±0.74	2.80±0.42	<0.0001	0.97±0.19	1.79±0.90	2.58±0.67	<0.0001	0.49±0.48	1.03±0.80	1.91±0.95	0.0003	0.32±0.48	1.05±0.85	2.30±0.62	<0.0001
**C**. Cell distribution	0.32±0.57	1.02±0.79	2.55±0.69	<0.0001	0.14±0.34	0.96±0.83	2.36±0.42	<0.0001	0.23±0.36	0.75±0.65	2.13±1.09	<0.0001	0.00±0.00	0.36±0.50	1.86±0.50	<0.0001
**D**. Cell population viability	3.00±0.00	3.00±0.00	3.00±0.00	N/A*	3.00±0.00	3.00±0.00	3.00±0.00	N/A*	2.81±0.75	3.00±0.00	3.00±0.00	0.6216	3.00±0.00	3.00±0.00	3.00±0.00	N/A*
**E**. Subchondral bone	2.35±0.99	2.30±1.16	3.00±0.00	0.1468	2.91±0.38	3.00±0.00	3.00±0.00	0.6216	2.19±1.33	2.31±1.16	2.53±1.06	0.8185	2.27±0.94	2.09±1.14	2.68±0.56	0.3032
**F**. Cartilage mineralization (calcified cartilage)	3.00±0.00	3.00±0.00	3.00±0.00	N/A*	3.00±0.00	3.00±0.00	3.00±0.00	N/A*	2.63±1.02	2.63±1.06	3.00±0.00	0.6012	3.00±0.00	3.00±0.00	3.00±0.00	N/A*
**G**. Type I collagen staining of the matrix	0.78±0.66	1.78±0.84	2.89±0.16	<0.0001	0.81±0.40	1.85±0.35	2.73±0.19	<0.0001	0.63±0.68	1.24±0.91	2.34±0.98	0.0002	0.27±0.55	0.82±0.72	1.71±0.85	<0.0001
**H**. Type II collagen staining of the matrix	1.14±0.59	2.00±0.64	2.81±0.18	<0.0001	1.11±0.27	2.10±0.37	2.73±0.33	<0.0001	0.96±0.72	1.30±0.77	2.56±1.04	0.0004	0.50±0.51	1.09±1.16	2.59±0.66	<0.0001

N/A*, *P* value not applicable because there were no variations in the measurements among the groups.

Supplementary Table 5. Gender differences in ICRS scores between male and female animals.

	Fibrin			eGFP			DCX-eGFP		
6-months rabbits	male	female	*P* value	male	female	*P* value	male	female	*P* value
Macroscopic score (mean ± SD)	7.50±1.31	6.63±2.48	0.17	8.08±3.20	8.67±1.03	0.69	11.83±0.41	11.80±0.24	0.87
Histological score (mean ± SD)	13.27±2.52	12.45±3.36	0.34	16.86±5.82	17.00±1.00	0.96	23.3±0.91	22.84±1.18	0.46
12-months rabbits	male	female	*P* value	male	female	*P* value	male	female	*P* value
Macroscopic score (mean ± SD)	7.75±1.42	6.71±3.00	0.20	9.25±0.76	8.47±1.58	0.23	11.73±0.28	11.38±0.78	0.41
Histological score (mean ± SD)	13.15±0.62	13.42±1.76	0.67	17.6±1.79	17.43±3.86	0.94	22.28±1.01	22.17±1.16	0.89
24-months rabbits	male	female	*P* value	male	female	*P* value	male	female	*P* value
Macroscopic score (mean ± SD)	6.96±2.38	6.20±2.35	0.46	8.33±2.80	7.40±3.65	0.65	11.83±0.41	11.82±0.20	0.94
Histological score (mean ± SD)	10.87±4.35	10.00±4.92	0.76	12.58±5.73	15.25±0.35	0.30	19.4±6.32	21.55±0.35	0.44
24-months monkeys	male	female	*P* value	male	female	*P* value	male	female	*P* value
Macroscopic score (mean ± SD)	5.92±3.09	6.30±2.67	0.75	9.00±1.41	6.66±5.03	0.36	11.50±1.22	11.20±1.10	0.67
Histological score (mean ± SD)	11.17±1.60	9.70±2.15	0.09	13.50±4.90	12.90±3.38	0.81	20.72±1.98	18.78±2.57	0.20

Supplementary Table 6. Animal health and cause of mortality.

	Total number	Healthy at endpoint	Died before endpoint	Abnormal necropsy	Main findings
Rabbits	42	40	2 (C117 and C5)	2 (C117 and C5)	C117 died at 2 months after surgery and C5 died at 18 months after surgery. Both animals showed gastrointestinal illness.
Monkeys	12	10	1 (HI72)	2 (HI72 and IA05)	HI72 showed lameness on the left leg at 9 months after surgery and swelling of the left knee and other joints developed over the next few weeks. HI72 failed to wake up after routine anesthesia and was humanely euthanized. Necropsy revealed polyarthritis, lymphadenopathy, and cachexia. IA05 survived until the endpoint of 24 months after surgery. Necropsy revealed severe hepatic amyloidosis, chronic renal interstitial fibrosis, and mild inflammation in the cecum.

Supplementary Table 7. Animal numbers and grouping.

	New Zealand White rabbits				Rhesus macaques
Endpoints	2 weeks	6 months	12 months	24 months	24 months
Animals at Surgery	5	13	12	12	12
Macroscopic analysis	5	12	12	11	11
Histological analysis	5	10	8	8	11
Reasons of exclusion from analyses		1 died at 2 months; 1 used for mechanical test; 1's samples damaged during sectioning	4 used for mechanical test	1 died at 18 months; 3 used for mechanical test	1 euthanized at 12 months

Supplementary Table 8. List of antibodies used.

Antigen	Host	Manufacturer	Catalog#	Lot#	Dilution
Aggrecan	mouse	abcam	ab3778	GR3235547-1	1:50
CD45	mouse	Thermo Fisher scientific	MA5-28392	WE3268002A	1:200
CD45	mouse	Thermo Fisher scientific	BDB552566	9301693	1:200
Collagen 1	mouse	Thermo Fisher scientific	MA1-26771	UC2725761	1:1500
Collagen 2	mouse	Thermo Fisher scientific	MA5-12789	VA2927282A	1:50
Collagen 10	mouse	Thermo Fisher scientific	50-112-2854	2338374	1:200
DCX	goat	Santa Cruz Biotechnology	sc-8066	B1116	Western blot: 1:500
DCX	mouse	Santa Cruz Biotechnology	sc-271390	D2720	Western blot: 1:500; IHC: 1:500
eGFP	mouse	Thermo Fisher scientific	MA1-952	VH307463	1:200
GDF5	mouse	Santa Cruz Biotechnology	sc-373744	A1719	1:200
GAPDH	mouse	Millipore Sigma	MAB374	3432602	1:5000
Ku80	mouse	Takara	Y40400	AK40008S	1:100
Matrilin 1	mouse	LifeSpan BioSciences	LS-C169264	62770	1:1000
Mitochondria	mouse	Millipore Sigma	MAB1273	3129248	1:100
Nuclei	mouse	Millipore Sigma	MAB1281	3189191	1:100
Matrilin 2	goat	R&D systems	AF3044	YII0119021	1:40
**Secondary antibodies and kits**	**Host**	**Manufacturer**	**Catalog#**		**Dilution**
VECTASTAIN Elite ABC HRP Kit (Peroxidase, mouse IgG)	Horse	VECTOR LABORATORIES	PK-6102	ZG0211	Per kit instructions
VECTASTAIN Elite ABC HRP Kit (Peroxidase, Goat IgG)	Rabbit	VECTOR LABORATORIES	PK-6105	ZF1024	Per kit instructions
Alexa Fluor 488-conjugated AffiniPure Donkey Anti-Goat IgG (H+L)	Donkey	Jackson ImmunoResearch Laboratories	705-545-147	136089	1:200
Rhodamine Red™-X-conjugated AffiniPure Donkey Anti-Mouse IgG (H+L)	Donkey	Jackson ImmunoResearch Laboratories	715-295-150	131606	1:200
