# Supplementary material for: Human adipose-derived stromal/stem cells expressing doublecortin improve cartilage repair in rabbits and monkeys
Source: NPJ Regen Med. 2021 Nov 30;6:82. doi: 10.1038/s41536-021-00192-6 (PMC8633050; doi:10.1038/s41536-021-00192-6)
Supplement: Supplementary file 2 — Reporting Summary [file 41536_2021_192_MOESM2_ESM.pdf]

nature research

Corresponding author(s): Zongbing You

Last updated by author(s): Oct 26, 2021

Reporting Summary

Nature Research wishes to improve the reproducibility of the work that we publish. This form provides structure for consistency and transparency in reporting. For further information on Nature Research policies, see our Editorial Policies and the Editorial Policy Checklist.

Statistics

For all statistical analyses, confirm that the following items are present in the figure legend, table legend, main text, or Methods section.

n/a	Confirmed	
☐	☒	The exact sample size (n) for each experimental group/condition, given as a discrete number and unit of measurement
☐	☒	A statement on whether measurements were taken from distinct samples or whether the same sample was measured repeatedly
☐	☒	The statistical test(s) used AND whether they are one- or two-sided<br>*Only common tests should be described solely by name; describe more complex techniques in the Methods section.*
☒	☐	A description of all covariates tested
☐	☒	A description of any assumptions or corrections, such as tests of normality and adjustment for multiple comparisons
☐	☒	A full description of the statistical parameters including central tendency (e.g. means) or other basic estimates (e.g. regression coefficient) AND variation (e.g. standard deviation) or associated estimates of uncertainty (e.g. confidence intervals)
☐	☒	For null hypothesis testing, the test statistic (e.g. F, t, r) with confidence intervals, effect sizes, degrees of freedom and P value noted<br>*Give P values as exact values whenever suitable.*
☒	☐	For Bayesian analysis, information on the choice of priors and Markov chain Monte Carlo settings
☒	☐	For hierarchical and complex designs, identification of the appropriate level for tests and full reporting of outcomes
☒	☐	Estimates of effect sizes (e.g. Cohen's d, Pearson's r), indicating how they were calculated

Our web collection on statistics for biologists contains articles on many of the points above.

Software and code

Policy information about availability of computer code

Data collection	No custom software or computer code was used.
Data analysis	Prism 8 software (GraphPad Software, San Diego, CA) was used for statistical analyses.

For manuscripts utilizing custom algorithms or software that are central to the research but not yet described in published literature, software must be made available to editors and reviewers. We strongly encourage code deposition in a community repository (e.g. GitHub). See the Nature Research guidelines for submitting code & software for further information.

Data

Policy information about availability of data

All manuscripts must include a data availability statement. This statement should provide the following information, where applicable:
- Accession codes, unique identifiers, or web links for publicly available datasets
- A list of figures that have associated raw data
- A description of any restrictions on data availability

All data associated with this study are present in the paper or the Supplementary Information. All relevant data are available from the authors.

Field-specific reporting

Please select the one below that is the best fit for your research. If you are not sure, read the appropriate sections before making your selection.

☒ Life sciences ☐ Behavioural & social sciences ☐ Ecological, evolutionary & environmental sciences

For a reference copy of the document with all sections, see nature.com/documents/nr-reporting-summary-flat.pdf

Life sciences study design

All studies must disclose on these points even when the disclosure is negative.

Sample size	The sample size (n=12 animals per time point per group) was determined by power analysis based on previously published studies.
Data exclusions	One rabbit in the 24-month follow up group was excluded from data analysis because the animal died at 18 months, prior to the endpoint; one monkey was excluded from data analysis because the animal was euthanized at 12 months due to illness, prior to the endpoint. Cartilage tissue samples used for mechanical tests were excluded from histological analysis due to tissue damages caused by mechanical tests. One animal's samples were damaged during tissue sectioning, thus being excluded from histological analysis.
Replication	In vitro experiments were replicated at least twice and biological replications (n) are shown in the figure legends.
Randomization	Animal's left or right knees were randomized into the treatment or control group through flipping a coin.
Blinding	Cartilage repair outcomes were evaluated in a blinded manner in which the evaluator was blinded to the grouping of the repaired cartilage defects.

Reporting for specific materials, systems and methods

We require information from authors about some types of materials, experimental systems and methods used in many studies. Here, indicate whether each material, system or method listed is relevant to your study. If you are not sure if a list item applies to your research, read the appropriate section before selecting a response.

Materials & experimental systems

n/a	Involved in the study	
☐	☒	Antibodies
☐	☒	Eukaryotic cell lines
☒	☐	Palaeontology and archaeology
☐	☒	Animals and other organisms
☒	☐	Human research participants
☒	☐	Clinical data
☒	☐	Dual use research of concern

Methods

n/a	Involved in the study	
☒	☐	ChIP-seq
☒	☐	Flow cytometry
☒	☐	MRI-based neuroimaging

Antibodies

Antibodies used	See Supplementary Table 8.
Validation	Validation was performed by the manufacturers (see the manufacturer's websites for relevant citations and antibody profiles). Quality control was included in the present study.

Eukaryotic cell lines

Policy information about cell lines

Cell line source(s)	Human adipose-derived stromal/stem cells (hASCs, passage zero) were provided by LaCell, LLC (New Orleans, LA)
Authentication	hASCs were characterized for immunophenotypes (CD29+CD34+CD73+CD90+CD105+CD44loCD45lo) and differentiation properties (adipogenesis and osteogenesis) .
Mycoplasma contamination	hASCs were tested negative for Mycoplasma contamination.
Commonly misidentified lines (See ICLAC register)	none.

Animals and other organisms

Policy information about studies involving animals; ARRIVE guidelines recommended for reporting animal research

Laboratory animals	42 New Zealand White rabbits (male: female = 1:1, retired breeders with estimated ages of about 1.5 years) were purchased from Charles River Laboratories (Wilmington, MA). 12 rhesus macaques (male: female = 1:1, average age 7.24 ± 1.37 years) were obtained from and housed at Tulane National Primate Research Center (Covington, LA).
Wild animals	None.
Field-collected samples	None.
Ethics oversight	All animal studies were approved by the Animal Care and Use Committee of Tulane University, which was in compliance with the U.S. Department of Health and Human Services Guide for the Care and Use of Laboratory Animals.

Note that full information on the approval of the study protocol must also be provided in the manuscript.
